# Dopamine D2 receptors bypass canonical signaling to directly tune NMDA receptor function and aversive learning

**DOI:** 10.1126/sciadv.aee6579

**Published:** 2026-07-23

**Authors:** Sheng Gong, Joy Adler, Ying Zhu, Charlize Szeto, Lisa Lin, Caroline Rauffenbart, Arturo Torres-Herraez, Wesley B. Asher, Jonathan A. Javitch, Christopher P. Ford

**Affiliations:** ^1^ Department of Pharmacology, University of Colorado School of Medicine, Anschutz Medical Campus, Aurora, CO 80045, USA.; ^2^ Howard Hughes Medical Institute, Janelia Research Campus, Ashburn, VA 20147, USA.; ^3^ Department of Psychiatry, Columbia University Vagelos College of Physicians & Surgeons, New York, NY 10032, USA.; ^4^ Division of Molecular Therapeutics, New York State Psychiatric Institute, New York, NY 10032, USA.; ^5^ Barnard College of Columbia University, New York, NY 10032, USA.; ^6^ Department of Molecular Pharmacology and Therapeutics, Columbia University Vagelos College of Physicians & Surgeons, New York, NY 10032, USA.

## Abstract

Dopamine D2 receptors (D2Rs) modulate reward learning and aversive behaviors, with dysfunction linked to addiction and psychiatric disorders. D2Rs regulate behavior through modulation of striatal glutamatergic transmission, yet how D2Rs control postsynaptic glutamate signaling remains poorly understood. Using molecular tools to selectively disrupt heteromeric interactions while preserving canonical signaling, we show that physical coupling between D2Rs and GluN2B-containing *N*-methyl-d-aspartate (NMDA) receptors in nucleus accumbens medium spiny neurons enables D2Rs to suppress NMDA receptor function independent of canonical G protein and arrestin signaling. This modulation was input specific, occurred at thalamic but not cortical synapses converging on the same neurons, constrained long-term potentiation, and, within the medial ventral nucleus accumbens, facilitated aversive learning. These findings reveal that D2Rs can bypass second messenger systems to tune glutamatergic transmission through receptor-receptor interactions, providing a mechanism by which dopamine selectively gates specific glutamatergic inputs to control striatal plasticity and behavioral adaptation.

## Teaser

Dopamine acts at D2-NMDA receptor heteromers to control glutamatergic transmission and aversive learning.

## INTRODUCTION

The nucleus accumbens (NAc) is densely innervated by both dopamine and glutamate inputs, which together play key roles in controlling the integration of motor behaviors with reward processing and motivation (*1*, *2*). Within the NAc, mesolimbic midbrain dopamine neurons from the ventral tegmental area (VTA) converge with glutamatergic inputs from the cortex, thalamus, amygdala, and hippocampus, with different striatal subregions receiving distinct combinations of glutamatergic and dopaminergic inputs due to the topographical variations in input projections (*3*–*7*). Acting as a dynamic modulator, dopamine shapes glutamate signaling in the NAc by gating inputs at both pre- and postsynaptic sites to regulate neuronal excitability, tune the strength of synaptic inputs, and govern the extent of excitatory synaptic plasticity (*8*–*13*). However, while disruptions in dopaminergic regulation of glutamate signaling are implicated in multiple psychiatric disorders, including substance use disorder and schizophrenia (*14*–*18*), the specific mechanisms by which D2 dopamine receptors differentially filter glutamatergic inputs to the accumbens remain poorly understood.

Dopamine D2 receptors (D2Rs) are Gα_i/o/z_-coupled receptors that are the major target of antipsychotic drugs currently used to treat schizophrenia (*19*, *20*). Through Gα, Gβγ, and β-arrestin signaling, D2Rs regulate multiple downstream targets including inhibition of adenylyl cyclase, activation of G protein–coupled inwardly rectifying potassium (GIRK) channels, and modulation of AKT/glycogen synthase kinase-3β (GSK-3β) signaling (*21*–*24*).

A major site of integration of glutamate and dopamine inputs in the NAc is within medium spiny neurons (MSNs), half of which express D2Rs. The location of D2R on dendritic shafts of MSNs and at glutamatergic afferent terminals (*25*–*30*) suggests that an important feature of D2R-mediated modulation is regulation of excitatory synaptic transmission. In addition to the inhibition of glutamate release by presynaptic D2Rs (*9*, *10*, *31*–*33*), D2Rs also regulate glutamatergic transmission through the modulation of *N*-methyl-d-aspartate (NMDA)–type glutamate receptors (NMDARs). Several mechanisms have been proposed, including cAMP (cyclic adenosine monophosphate)–dependent protein kinase (PKA)–dependent modulation of Ca^2+^ influx and GSK-3β–dependent modulation of NMDAR currents (*33*, *34*). In addition, D2Rs have been reported to heterodimerize with NMDARs through direct interaction between the N-terminal portion of the third intracellular loop (IL3) of D2R and the C-terminal tail of the GluN2B subunit of the NMDAR (*35*, *36*). However, while D2R activation was shown to inhibit NMDAR currents in dissociated striatal neurons through this interaction (*35*), postsynaptic modulation of NMDA currents by D2Rs was not observed when examining synaptic NMDARs through glutamate uncaging onto dendritic spines in striatal slices (*33*). This discrepancy has left the mechanism and functional role of D2R-NMDAR interactions unresolved for nearly two decades.

This discrepancy became more acute when it was demonstrated that D2R-NMDAR heteromers are nonetheless behaviorally relevant: An interfering peptide that disrupts the interaction blocked cocaine-induced locomotor sensitization, conditioned place preference, and its reinstatement, while preserving natural reward processing (*36*). However, if D2Rs do not modulate NMDARs at synapses, then how could heteromers control behavior? Three critical questions emerged. First, what is the signaling mechanism that links D2R activation to NMDAR modulation? Second, is this modulation input-specific? Third, what is the physiological function of these heteromers beyond cocaine-related behaviors?

Here, we investigated the mechanisms by which D2Rs modulate postsynaptic NMDARs in MSNs of the NAc and dorsal striatum (DStr). We developed molecular tools—a Cre-dependent minigene encoding the critical D2R IL3 sequence (T225-A234) and a D2R deletion mutant [D2R(Δ225 to 234)]—that selectively disrupt heteromeric interactions while preserving canonical D2R signaling. Using these tools in ex vivo slice electrophysiological recordings, combined with pharmacological and genetic approaches, we found that D2Rs reduced postsynaptic NMDAR currents through a physical coupling between IL3 of D2R and GluN2B-containing NMDARs, independent of G protein and arrestin signaling. This modulation was input specific, occurring at thalamic but not cortical synapses converging on the same neurons—directly resolving the discrepancy with previous uncaging studies, where glutamate release at spine heads activated NMDARs not in proximity to dendritic D2Rs. This interaction regulated the induction of long-term synaptic potentiation (LTP) and, within the medial ventral NAc, facilitated aversive learning. Together, these results indicate that D2Rs can bypass canonical signaling cascades to directly modulate glutamatergic transmission through physical coupling with NMDARs, providing a distinct mechanism for input-selective neuromodulation in the NAc.

## RESULTS

### NAc D2Rs selectively inhibit GluN2B-containing NMDAR currents

**Fig. 1.**
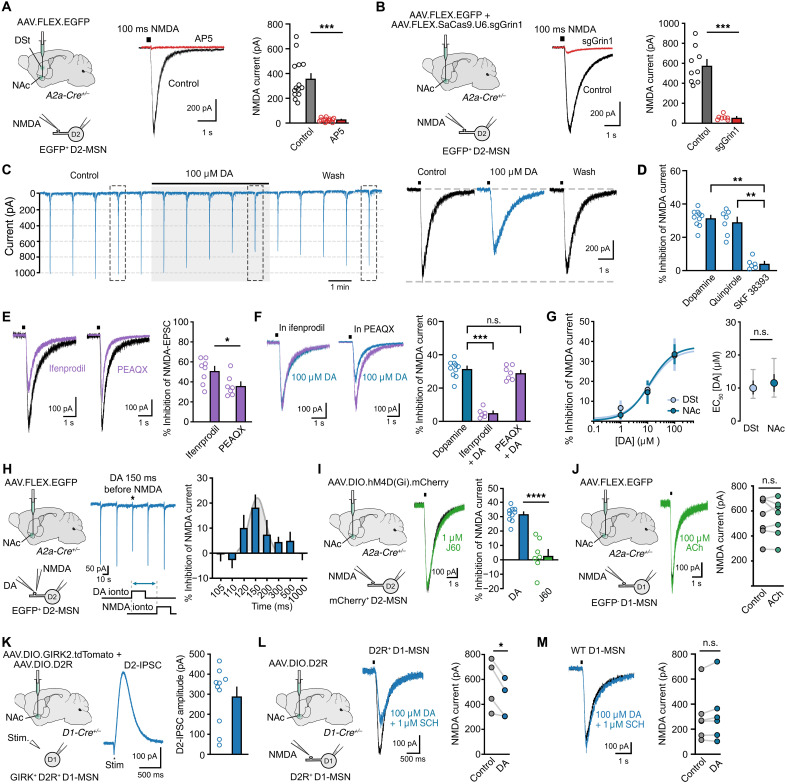
D2R modulation of GluN2B-containing NMDARs. (**A**) EGFP expression in NAc D2-MSNs and NMDA currents blocked by AP5 (50 μM). *n* (cells)/*N* (animals) = 14/5. (**B**) NMDAR currents from control (single-guide RNA targeting *Grin1*; sg*Grin1*^*−*^) and *Grin1* knockout (sg*Grin1*^*+*^) neurons. Control, *n*/*N* = 9/2; sg*Grin1*, *n*/*N* = 7/2. (**C**) Dopamine (DA; 100 μM) inhibition of NMDA current in EGFP^+^ D2-MSNs. (**D**) Dopamine (100 μM; *n*/*N* = 10/5), quinpirole (1 μM; *n*/*N* = 7/4), and SKF38393 (10 μM; *n*/*N* = 6/3) effects on NMDA currents. (**E**) Ifenprodil (3 μM; *n*/*N* = 8/4) and PEAQX (0.1 μM; *n*/*N* = 7/4) effects on NMDA currents. (**F**) Dopamine (100 μM) effects on NMDA currents in ifenprodil (*n*/*N* = 6/4) or PEAQX (*n*/*N* = 7/4). (**G**) Dopamine concentration-response relationships for D2R inhibition of NMDA currents in the DStr (*n*/*N* = 14/5) and NAc (*n*/*N* = 17/5) and EC_50_ values (gray traces represent 95% confidence intervals). (**H**) Inhibition of NMDA currents by iontophoretic (ionto) application of dopamine. *n*/*N* = 3 to 8/5. (**I**) No effect of JHU37160 (1 μM) on NMDA currents from hM4D(Gi)-expressing D2-MSNs. *n*/*N* = 6/3. (**J**) No effect of acetylcholine (ACh) (100 μM; with 100 nM ambenonium) on NMDA currents in D1-MSNs. *n*/*N* = 7/4. (**K**) Injection of AAV.DIO.GIRK2 and AAV.DIO.D2R in D1-Cre mice. Electrically evoked D2-IPSCs in D2R-expressing D1-MSNs. *n*/*N* = 10/3. (**L**) Dopamine [100 μM; in the presence of 1 μM SCH23390 (SCH)] inhibition of NMDA currents in D2R-expressing D1-MSNs. *n*/*N* = 4/2. (**M**) Lack of effect of dopamine (100 μM; in 1 μM SCH23390) on NMDA currents in wild-type (WT) control (D2R^−^) D1-MSNs. *n*/*N* = 6/2. Dopamine inhibition of NMDA current data is shown [(D), (F), and (I)]. Summary data are means ± SEM. n.s., *P* > 0.05; **P < 0.01; ***P < 0.001; *P < 0.05; ****P < 0.0001. Statistical data and tests are provided in table S1.

To determine how D2Rs modulate NMDAR signaling, we made voltage clamp recordings from enhanced green fluorescent protein–expressing (EGFP^+^) D2-MSNs in the NAc medial shell of *Adora2a*-Cre (A2a-Cre^+/−^) mice, 3 weeks following injection of a Cre-dependent adeno-associated virus (AAV)–encoding EGFP. We began by locally applying NMDA via an iontophoretic pipette positioned near a D2-MSN to isolate postsynaptic responses and bypass presynaptic D2R effects on glutamate release (*10*, *31*, *33*). Using an artificial cerebrospinal fluid (aCSF) lacking extracellular Mg^2+^ and containing antagonists for AMPA and γ-aminobutyric acid type A (GABA_A_) receptors, local application of NMDA evoked inward currents in D2-MSNs that were abolished by the NMDAR antagonist D-AP5 (50 μM) (Fig. 1A) or by genetic inactivation of *Grin1*, which encodes the essential GluN1 subunit of the NMDAR, following injection of a Cre-dependent CRISPR Staphylococcus aureus Cas9 (SaCas9) vector (Fig. 1B). Bath application of either dopamine (100 μM) or the D2-type dopamine receptor agonist quinpirole (1 μM) reduced the amplitude of NMDA-mediated currents, while application of the D1-type dopamine receptor agonist SKF38393 (10 μM) had no effect (Fig. 1, C and D). As striatal NMDARs are thought to be primarily composed as diheteromeric tetramers (GluN1/GluN2A or GluN1/GluN2B) (*37*–*39*) and the GluN2 subunit composition determines the physiological properties of NMDARs (*40*), we examined their contributions to NMDAR currents in D2-MSNs and the receptor subtypes involved in D2R-dependent modulation. Inhibition of GluN2B-containing NMDARs with ifenprodil (3 μM) reduced NMDAR–excitatory postsynaptic currents (EPSCs) by ~50%, while the GluN2A blocker PEAQX (NVP-AAM077) (0.1 μM) caused a ~40% reduction (Fig. 1E). However, D2R-dependent modulation was occluded only in the presence of ifenprodil and not PEAQX (Fig. 1F), indicating that D2R-mediated inhibition of NMDARs requires GluN2B-containing NMDARs.

As the sensitivity of intracellular D2R signaling differs between the DStr and NAc due to differences in Gα_i_ versus Gα_o_ coupling (*41*, *42*), we generated dose-response curves to examine whether the sensitivity of D2R-mediated inhibition of NMDA currents also differs across these two regions. The data, however, revealed a similar half-maximal effective concentration (EC_50_) of dopamine for inhibition in both the dorsal medial striatum and NAc shell (Fig. 1G). Thus, despite D2Rs having differing sensitivity in G protein coupling in these two regions (*41*, *42*), D2Rs appear to have similar potency and efficacy across striatal regions for inhibiting NMDARs.

To determine the time course over which D2Rs inhibit NMDAR currents, we sequentially applied dopamine and NMDA, using two iontophoretic pipettes placed near a D2-MSN. Varying the duration between dopamine and NMDA iontophoresis revealed that applying dopamine ~150 ms before NMDA led to maximal inhibition of NMDA currents (Fig. 1H), indicating that this modulation occurs rapidly.

We next investigated whether other Gα_i/o_-coupled G protein–coupled receptors (GPCRs) also modulate NMDAR currents in D2-MSNs, as might be expected whether the effect were mediated by G protein–dependent signaling. We first examined chemogenetic designer receptors exclusively activated by designer drugs (DREADDs). Three weeks following viral expression of the Gα_i_-coupled human M4 muscarinic DREADD (designer receptor exclusively activated by designer drugs) (hM4Di) in NAc D2-MSNs, we found that unlike dopamine (100 μM), application of the DREADD agonist JHU37160 (1 μM) had no effect on the amplitude of NMDAR currents (Fig. 1I). Next, we examined muscarinic receptors in D1-MSNs, which express both Gα_q_-coupled M1 receptors and Gα_i/o_-coupled M4 receptors (*43*, *44*). Here, application of acetylcholine (100 μM) also had no effect on the amplitude of NMDAR currents (Fig. 1J). Last, as D1-MSNs lack D2R, we further confirmed the specific ability of D2Rs to modulate NMDAR currents by ectopically expressing D2Rs in D1-MSNs by viral injection of AAV.DIO.D2R.EGFP into the NAc of D1-Cre^+/−^ mice. To confirm that D2Rs were functional in these cells, we coinjected an AAV-expressing GIRK channels (AAV.DIO.GIRK2.tdTomato), which are efficiently activated by D2Rs in D2-MSNs via Gβγ subunits, to provide an electrophysiological readout of D2R-mediated G protein signaling (*41*, *42*, *45*). In D2R^+^ GIRK2^+^ D1-MSNs, a D2R-dependent inhibitory synaptic current (D2-IPSC) was observed after evoking dopamine release, confirming that ectopically expressed D2Rs in D1-MSNs were functional (Fig. 1K). Examining the effect of dopamine on NMDA currents in D2R^+^ D1-MSNs revealed that application of dopamine (100 μM; in the presence of the D1R antagonist SCH23390, 1 μM) was able to evoke a robust inhibition of NMDA currents in all four cells tested (Fig. 1L), an effect that was not seen in control D1-MSNs lacking D2Rs (Fig. 1M). Together, these findings indicate that D2Rs selectively inhibit GluN2B-containing NMDAR currents in NAc D2-MSNs and can also do so when expressed in other neurons.

### D2R-mediated inhibition of NMDAR currents is independent of G protein and arrestin-mediated signaling

**Fig. 2.**
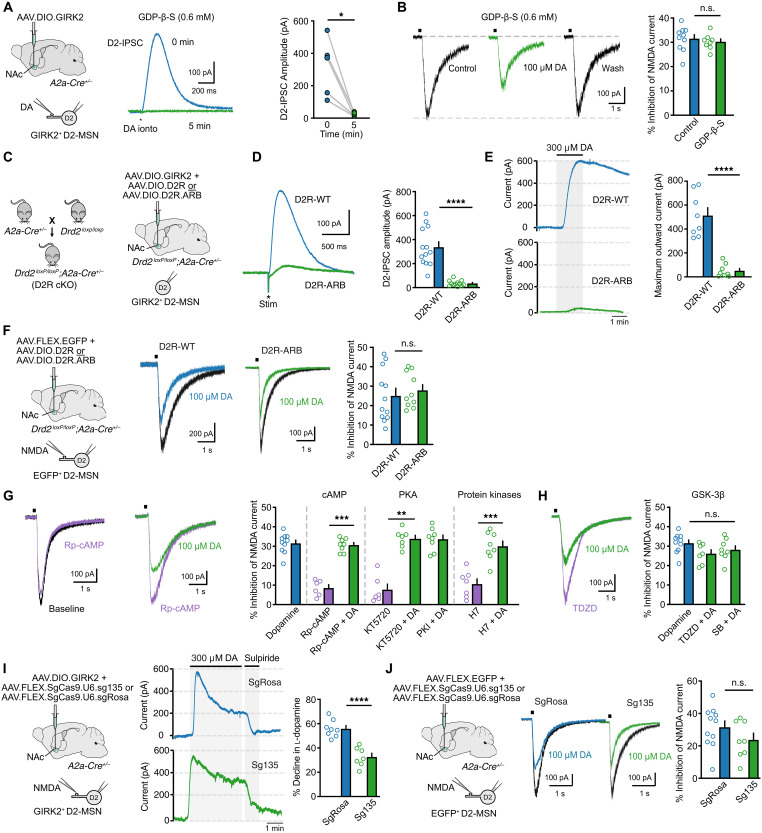
D2R inhibition of NMDAR currents is independent of G protein, arrestin, and kinase signaling. (**A**) Block of D2-IPSCs following GDP-β-S dialysis (0.6 mM). *n*/*N* = 5/2. (**B**) No effect of GDP-β-S (0.6 mM; 15 min) on dopamine (DA) inhibition of NMDA currents. Control, *n* = 10; GDP-β-S, *n* = 8/3. (**C**) Expression of GIRK2 and D2R-WT or D2R-ARB in D2R cKO mice. (**D**) D2-IPSCs from D2-MSNs expressing D2R-WT (*n*/*N* = 13/4) or D2R-ARB (*n*/*N* = 12/4). (**E**) D2R currents from D2-MSNs expressing D2R-WT (*n*/*N* = 8/4) or D2R-ARB (*n*/*N* = 8/4). (**F**) Dopamine inhibition of NMDA currents from D2-MSNs expressing D2R-WT (*n*/*N* = 12/4) or D2R-ARB (*n*/*N* = 10/4). (**G**) Lack of effect of Rp-cAMPS (10 μM), KT5720 (10 μM), PKI (30 μM) and H7 (30 μM) on dopamine inhibition of NMDA currents. Rp-cAMPS, *n*/*N* = 6/5; Rp-cAMPS + DA, *n*/*N* = 8/5; KT5720, *n*/*N* = 6/4; KT5720 + DA, *n*/*N* = 7/4; PKI + DA, *n*/*N* = 8/3; H7, *n*/*N* = 7/3; H7 + DA, *n*/*N* = 8/3. (**H**) Lack effect of TDZD (10 μM; *n*/*N* = 7/4) and SB216763 (10 μM; *n*/*N* = 8/4) on dopamine inhibition of NMDA currents. (**I**) Reduced desensitization of D2R currents in arrestin knockdown (sg135) but not control (sg*Rosa*) cells. DA, 300 μM; sulpiride, 3 μM. sg*Rosa*, *n*/*N* = 8/4; Sg135, *n*/*N* = 7/4. (**J**) Lack of effect of arrestin knockdown (sg135) on dopamine inhibition of NMDA currents compared to control (sg*Rosa*) cells. sg*Rosa*, *n*/*N* = 11/4; Sg135, *n*/*N* = 8/4. Control data for dopamine inhibition of NMDA currents in (B), (G), and (H) are from Fig. 1. Summary data are means ± SEM. n.s., *P* > 0.05; **P < 0.01; ***P < 0.001; *P < 0.05; ****P < 0.0001. Statistical data and tests are provided in table S1.

To investigate the mechanism by which D2Rs modulate NMDAR currents in D2-MSNs, we next examined the downstream pathways involved. GPCRs, such as D2Rs, canonically modulate intracellular activity through the activation of G protein–mediated second messenger signaling and the recruitment of arrestin (*21*, *22*). To test for the necessity of G protein signaling, we replaced guanosine triphosphate (GTP) in the intracellular recording solution with the guanosine diphosphate (GDP) analog guanosine 5′-*O*-(2′-thiodiphosphate) (GDP-β-S; 0.6 mM), which acts as a competitive antagonist for GTP binding, thereby preventing G protein signaling. As a control, we first confirmed that GDP-β-S effectively blocked D2R-mediated G protein signaling by again virally expressing GIRK2 channels in D2-MSNs. Three weeks after viral injection, recordings from GIRK2^+^ D2-MSNs showed that dialysis with GDP-β-S (0.6 mM) eliminated dopamine-evoked D2R-mediated GIRK currents, confirming inhibition of G protein signaling (Fig. 2A). In contrast, dialysis of D2-MSNs with GDP-β-S failed to prevent dopamine (100 μM) from reducing the amplitude of NMDAR currents (Fig. 2B), suggesting that D2R-mediated inhibition of NMDAR currents does not require G protein activation. To further confirm a lack of involvement of G proteins, we next used D2R(L123W:R132L), an arrestin-biased mutant (D2R-ARB) that maintains robust recruitment of arrestin while showing markedly impaired G protein activation (*46*). We expressed this arrestin-biased mutant in D2-MSNs by injecting a Cre-dependent AAV encoding D2R-ARB into the NAc of D2R conditional knockout (*Drd2*^*loxp/loxp*^; A2a-Cre^+/−^) (D2R cKO) mice (Fig. 2C). As a control, we used a Cre-dependent AAV encoding wild-type D2Rs (D2R-WT) to reexpress control D2Rs back into D2-MSNs in these D2R cKO mice. We first confirmed that D2R-ARB had impaired G protein activation by measuring activation of GIRK2 channels. In MSNs expressing D2R-WT, both electrical stimulation and application of a saturating concentration of dopamine (300 μM) (*42*) produced robust GIRK channel–mediated D2-IPSCs and outward currents, which were absent in MSNs expressing D2R-ARB (Fig. 2, D and E), confirming the reduced ability of D2R-ARB to activate G proteins. In contrast, when tested for their effects on modulating NMDARs, we found that both D2R-WT and D2R-ARB were equally effective in reducing the amplitude of NMDAR currents (Fig. 2F). Together, these results suggest that D2R inhibition of NMDAR currents in D2-MSNs occurs independently of G protein activation.

NMDARs are regulated by various protein kinases, including PKA and protein kinase C (PKC), which can phosphorylate the receptor to modulate Ca^2+^ influx (*33*, *47*, *48*). However, bath application of Rp-cAMPS (10 μM), a cAMP analog that blocks PKA activation, had no basal effect on NMDAR currents and failed to prevent dopamine from reducing the amplitude of NMDAR currents (Fig. 2G). We also bath applied a PKA inhibitor (KT5720, 10 μM). The inhibitor alone had no basal effect on NMDA currents and also failed to block dopamine suppression of their amplitude (Fig. 2G), as did the PKA inhibitor, PKI (30 μM) (Fig. 2G). Thus, consistent with our findings of the lack of dependence on G protein signaling, these results demonstrate that downstream PKA signaling is also not involved in D2R-mediated modulation of NMDAR currents. Last, to explore whether other kinases might contribute, we applied the broad-spectrum serine/threonine kinase inhibitor, H7 (30 μM). It also had no basal effect or effect on dopamine-induced inhibition of NMDAR currents (Fig. 2G).

Beyond classical G protein signaling, D2R activation recruits arrestin, which terminates G protein signaling, facilitates receptor internalization, and initiates noncanonical G protein–independent pathways (*46*, *49*, *50*). Previous studies have demonstrated that D2R activation can recruit GSK-3β via an upstream arrestin3/protein phosphatase 2A pathway (*34*, *51*). In the prefrontal cortex, recruited GSK-3β has been shown to phosphorylate β-catenin, enhancing its interaction with GluN2B, potentially linking D2R-arrestin signaling to NMDAR modulation (*34*). To test whether this pathway is involved in D2R-mediated regulation of NMDAR currents in the NAc, we examined two separate GSK-3β inhibitors, TDZD (10 μM) and SB216763 (10 μM). Neither inhibitor had an effect on basal NMDAR currents, nor did they block dopamine-induced suppression of their amplitude (Fig. 2H). To further confirm these findings and minimize potential off-target effects from pharmacological agents, we used CRISPR-Cas9 to knock down β-arrestin2 specifically in D2-MSNs. We designed an AAV vector containing an inverted SaCas9 sequence flanked by loxP sites for Cre-mediated inversion, alongside a U6 promoter driving single guide RNA (sgRNA) expression targeting β-arrestin2 (AAV.FLEX.SaCas9.U6.sgRNA). As a control, we included a nontargeting sgRNA aimed at intron 1 of the *Rosa26* locus, which does not affect neuronal function (*52*, *53*). To assess the efficiency of SaCas9-mediated mutagenesis in D2-MSNs, we coinjected AAV.DIO.GIRK2.tdTomato with AAV.FLEX.SaCas9.U6.sg135 or sg*Rosa*. Four weeks postinjection, electrical stimulation in the striatum evoked D2-IPSCs with expected kinetics and amplitude (fig. S1). The rate of D2R desensitization, as measured by a reduction in GIRK current amplitude in response to a saturating concentration of dopamine (300 μM), was reduced in D2-MSNs expressing sg135 when compared to sg*Rosa*, indicating successful knockdown of arrestin function (Fig. 2I). Examining the effect of dopamine on NMDAR currents in arrestin knockdown D2-MSNs revealed a similar degree of NMDAR inhibition as in control cells, indicating that arrestin signaling is not required for D2R-mediated inhibition of NMDARs (Fig. 2J). Collectively, our data from pharmacological inhibition and CRISPR-Cas9 approaches indicate that D2R-mediated inhibition of NMDAR currents in D2-MSNs is independent of G protein, arrestin, cAMP, PKA, PKC, and GSK-3β signaling.

### Direct NMDAR current modulation by D2Rs

**Fig. 3.**
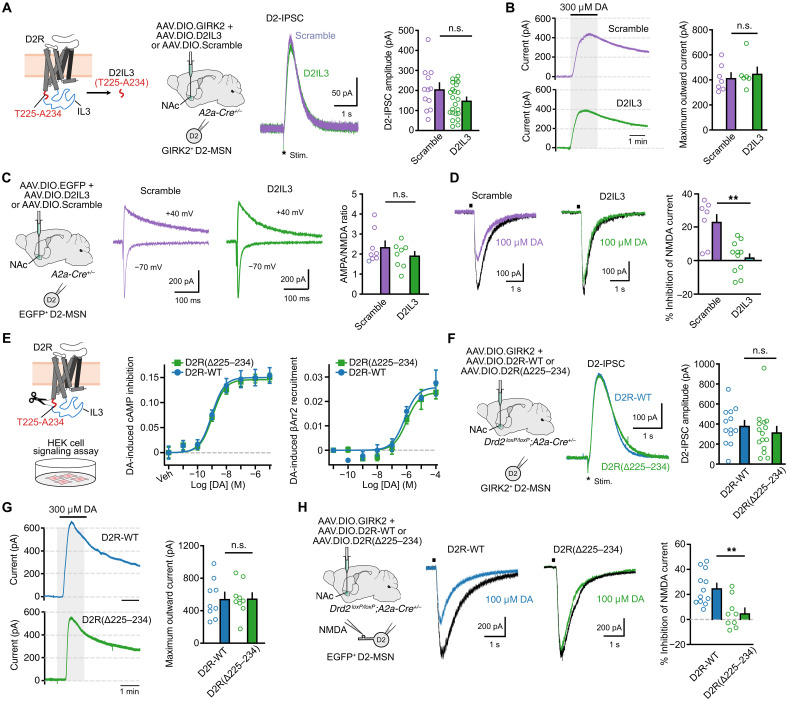
D2Rs modulate NMDA currents through an interaction involving IL3. (**A**) Schematic of minigene of T225-A234 of D2R IL3 and injection of AAV.DIO.GIRK2 and AAV.DIO.D2IL3 or AAV.DIO.scramble in A2a-Cre mice and recordings of evoked D2-IPSCs from D2-MSNs expressing D2R IL3 or scrambled sequence. Scramble, *n*/*N* = 11/4; D2IL3, *n*/*N* = 22/4. (**B**) GIRK currents evoked by application of dopamine (DA; 300 μM) from D2-MSNs expressing D2R IL3 or scrambled sequence. Scramble, *n*/*N* = 7/4; D2IL3, *n*/*N* = 6/4. (**C**) Electrically evoked AMPA- and NMDA-EPSCs from D2-MSNs expressing D2R IL3 or scrambled sequence. Scramble, *n*/*N* = 8/3; D2IL3, *n*/*N* = 8/3. (**D**) Dopamine modulation of NMDA currents in D2-MSNs expressing D2R IL3 or scrambled sequence. Scramble, *n*/*N* = 7/5; D2IL3, *n*/*N* = 11/5. (**E**) Schematic of D2R(Δ225 to 234) illustrating truncation of T225-A234 of D2R IL3. Dopamine-induced cAMP inhibition and β-arrestin2 (βarr2) recruitment assays performed in HEK cells. Three independent experiments were performed with triplicate samples. (**F**) Injection of AAV.DIO.GIRK2 and AAV.DIO.D2R-WT or AAV.DIO.D2R(Δ225 to 234) in D2R cKO (*Drd2*^*loxp/loxp*^; A2a-Cre ^+/−^) mice and evoked D2-IPSCs from D2-MSNs expressing D2R-WT or D2R(Δ225 to 234). D2R-WT, *n*/*N* = 13/4; D2R(Δ225 to 234), *n*/*N* = 15/4. (**G**) GIRK currents evoked by application of dopamine (300 μM) from D2-MSNs expressing D2R-WT or D2R(Δ225 to 234). D2R-WT, *n*/*N* = 9/4; D2R(Δ225 to 234), *n*/*N* = 9/4. (**H**) Dopamine modulation of NMDA currents in EGFP^+^ D2-MSNs expressing D2R-WT or D2R(Δ225 to 234). D2R-WT, *n*/*N* = 12/4; D2R(Δ225 to 234), *n*/*N* = 9/4. Summary data are means ± SEM. n.s., *P* > 0.05; **P < 0.01. Extended statistical data and tests are provided in table S1.

D2Rs have been shown to interact with NMDARs via the C-terminal tail of the GluN2B subunit of the NMDAR and the IL3 of D2R. Residues critical for this interaction have been mapped to the sequence T225-A234 (TKRSSRAFRA) within D2R IL3, and a peptide bearing this sequence has been shown to disrupt the interaction of D2R and NMDAR (*36*). To assess the role and consequence of this interaction in intact NAc circuits, we generated a Cre-dependent minigene encoding this D2R IL3 sequence and expressed it in D2-MSNs (Fig. 3A). We first confirmed that expression of the D2R IL3 minigene did not interfere with D2R–G protein signaling by measuring D2R-mediated GIRK currents and found no difference in the amplitude or kinetics of GIRK-mediated D2-IPSCs in cells expressing either the D2R IL3 sequence or a scrambled control sequence nor a difference in the amplitude of outward currents evoked by application of a saturating concentration of dopamine (300 μM) (Fig. 3, A and B, and fig. S2). To confirm that expression of the D2R IL3 minigene had no basal effect on NMDAR function, we electrically evoked glutamate release to record AMPA receptor (AMPAR)– and NMDAR-mediated EPSCs. Comparing AMPAR and NMDAR EPSCs revealed a similar AMPAR/NMDAR ratio in cells expressing either the D2R IL3 or scrambled sequence, suggesting that intrinsic NMDAR function was unaffected by expression of the minigene (Fig. 3C). In contrast, we found that expression of the D2R IL3 peptide prevented dopamine from inhibiting NMDA currents (Fig. 3D), suggesting that D2R modulation of NMDAR currents relies on this D2R IL3-GluN2B interaction.

To further confirm the role of interactions between D2R IL3 and GluN2B, we created a D2R(Δ225 to 234) deletion variant lacking the critical NMDAR-interacting sequence within IL3. In human embryonic kidney (HEK) 293 cells, this construct inhibited cAMP and recruited arrestin3 in a WT manner (Fig. 3E). Expression of D2R(Δ225 to 234) in D2-MSNs of D2R cKO mice (*Drd2*^*loxp/loxp*^; A2a-Cre^+/−^) restored normal G protein activation, as assessed by the ability to activate GIRK currents (Fig. 3, F and G, and fig. S2). However, unlike D2R-WT, D2R(Δ225 to 234)-expressing D2-MSNs failed to show an inhibition of NMDAR currents following dopamine application (Fig. 3H). Thus, while D2R-ARB—which lacks G protein signaling but retains the IL3 domain—inhibits NMDAR currents normally, D2R(Δ225 to 234)—which retains canonical signaling but lacks the IL3 domain—does not. Together with the GDP-β-S and arrestin knockdown experiments, these results establish that D2Rs rapidly modulate NMDAR currents through a physical coupling of IL3-GluN2B rather than canonical signaling cascades. Although purified fragments of D2R IL3 and the C terminus of GluN2B have been shown to interact directly (*35*), we cannot rule out a role for additional proteins that may be involved in the functional regulation of the D2R-NMDAR complexes in a cellular context, although the IL3-GluN2B interface is required.

### Enhancing tonic dopamine levels facilitates D2R inhibition of NMDAR signaling

**Fig. 4.**
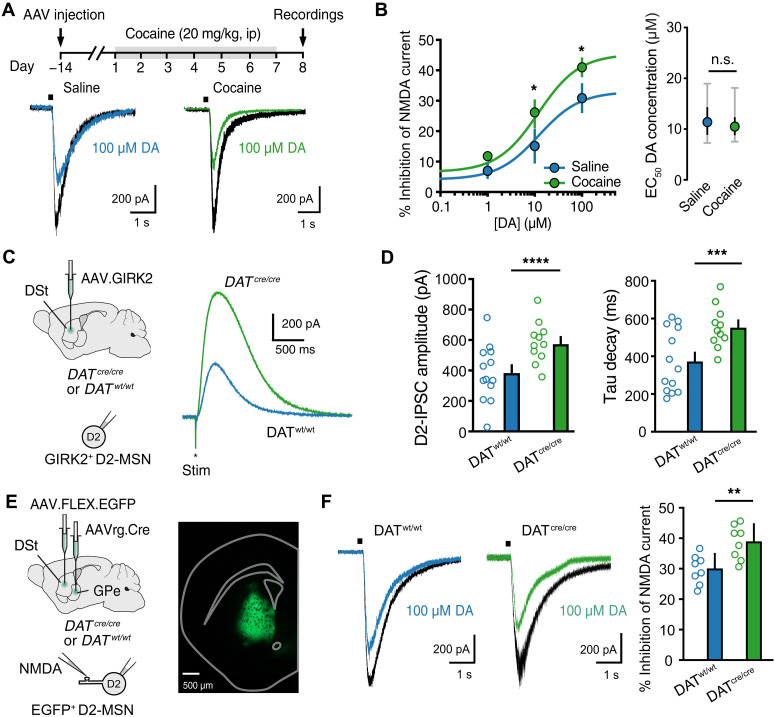
Enhancing dopamine tone increases D2R inhibition of NMDAR currents. (**A**) Timeline of AAV injection and chronic cocaine administration. Two weeks following AAV injection, mice were treated with cocaine (20 mg/kg, ip) or saline for 7 days and recordings made on the 8th day. Bottom: Representative recordings showing dopamine (DA) modulation of NMDA currents in EGFP^+^ D2-MSNs from saline- or cocaine-treated animals. (**B**) Concentration-response curves and EC_50_ values for dopamine inhibition of NMDAR currents following 7-day treatment of cocaine (20 mg/kg) or saline. Saline, *n*/*N* = 16/4; cocaine, *n*/*N* = 18/5. (**C**) Striatal injection of AAV.DIO.GIRK2 into C57Bl6 control (DAT^wt/wt^) or DAT*^cre/cre^* mice and recordings of evoked D2-IPSCs from D2-MSNs. (**D**) Summary of the D2-IPSCs amplitude and tau decay from control or DAT*^cre/cre^* mice. DAT^wt/wt^, *n*/*N* = 13/3. DAT*^cre/cre^*, *n*/*N* = 11/4. (**E**) Schematic of the injection of AAVrg.hSyn.Cre into the GPe and AAV9.FLEX.EGFP into the DStr of control or DAT*^cre/cre^* mice. Right: Enhanced yellow fluorescent protein (EYFP) fluorescence in the DStr. (**F**) Dopamine modulation of NMDA currents in EGFP^+^ D2-MSNs from control or DAT*^cre/cre^* mice. DAT^wt/wt^, *n*/*N* = 8/2; DAT*^cre/cre^*, *n*/*N* = 8/3. Summary data are means ± SEM. n.s., *P* > 0.05; **P < 0.01; ***P < 0.001; *P < 0.05; ****P < 0.0001. Extended statistical data and tests are provided in table S1.

Past work has found that repeated dosing of cocaine enhances the proportion of heterodimers formed between D2R and NMDAR GluN2B subunits in the striatum (*36*). To determine whether this increase in heteromers results in a greater inhibition of NMDAR by dopamine, we treated mice with cocaine [20 mg/kg, intraperitoneally (ip)] daily for 7 days to elevate mesolimbic dopamine levels in vivo. The following day recordings were made from D2-MSNs in brain slices containing NAc (Fig. 4A), and dopamine concentration-response relationships were generated to examine D2R-mediated inhibition of NMDAR currents. Following chronic cocaine treatment, we found that dose-response curves were shifted upward but had no change in their EC_50_ values when compared to saline treated controls (Fig. 4, A and B), indicating that chronic exposure to cocaine leads to an enhancement in the extent to which D2Rs inhibit NMDA currents.

To further evaluate whether a sustained increase in dopamine tone promotes D2R-NMDAR modulation, we generated homozygous DAT^*IREScre/IREScre*^ mice, which exhibit a 47% reduction in dopamine transporter (DAT) levels relative to WT mice (*54*). This DAT reduction impairs dopamine uptake, resulting in elevated dopamine levels following release (*54*, *55*), which could be seen by an increase in the amplitude and decay time of electrically evoked GIRK2-mediated D2-IPSCs in homozygous mice (Fig. 4, C and D). Because these mice lacked Cre-recombinase in D2-MSNs, we injected a retrograde AAVrg-Cre into the external segment of the globus pallidus (GPe) and a Cre-dependent AAV.FLEX.EGFP into the DStr of DAT^*IREScre/IREScre*^ or control littermate (DAT^wt/wt^) mice to label “indirect” pathway D2-MSNs of the DStr (Fig. 4E). These experiments were done in the DStr as both D1-MSNs and D2-MSNs in the NAc project to the ventral mesencephalon and the ventral pallidum (*56*), limiting our ability to identify NAc D2-MSNs based on target projections. Using this approach, we found that the extent of inhibition of NMDA currents by dopamine was enhanced in DStr D2-MSNs from DAT^*IREScre/IREScre*^ mice compared to littermate controls (Fig. 4F). These findings suggest that a persistent elevation in dopamine tone enhances the functional extent of D2R-NMDAR modulation.

### D2R modulation of NMDAR currents is synapse specific

**Fig. 5.**
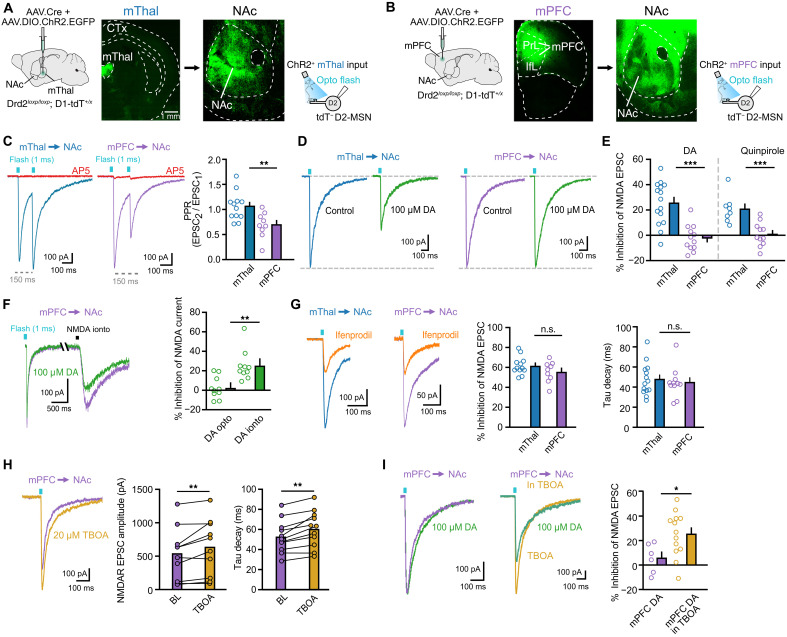
D2Rs modulate NMDARs in an input-specific manner. (**A**) mThal injection of AAV.Cre and AAV.DIO.ChR2.EGFP in *Drd2*^*loxp/loxp*^; D1-tdTomato^+/−^ (tdT^+/−^) mice showing EGFP expression at injection site (mThal) and projections to NAc. Cortex (CTx). (**B**) mPFC injection of AAV.Cre and AAV.DIO.ChR2.EGFP in *Drd2*^*loxp/loxp*^; D1-tdTomato^+/−^ mice showing EGFP expression at injection site (mPFC) and projections to NAc. Prelimbic cortex (PrL); Infralimbic cortex (IfL). (**C**) NMDA EPSCs from mThal or mPFC inputs in tdTomato^+^ putative NAc D2-MSNs, and paired-pulse ratios (PPR) mThal, *n*/*N* = 12/2; mPFC, *n*/*N* = 9/3. (**D**) Dopamine (DA) modulation of NMDA EPSCs from mThal but not mPFC inputs. (**E**) Quantification of inhibition of NMDA EPSCs by dopamine (100 μM) and quinpirole (1 μM). mThal + DA, *n*/*N* = 15/4; mPFC + DA, *n*/*N* = 12/5; mThal + quinpirole, *n*/*N* = 8/2; mPFC + quinpirole, *n*/*N* = 11/6. (**F**) Dopamine modulation of currents evoked by exogenous application of NMDA but not EPSCs evoked from mPFC inputs. *n*/*N* = 10/4. (**G**) Effect of ifenprodil (3 μM) on NMDA EPSCs from mThal (*n*/*N* = 12/4) and mPFC (*n*/*N* = 9/5) inputs. Right: Quantification of time course of decay of control NMDA EPSCs in the absence of ifenprodil. mThal, *n*/*N* = 14/4; mPFC, *n*/*N* = 11/4. (**H**) Potentiation of the amplitude and decay time of NMDA EPSCs evoked from mPFC inputs by TBOA (20 μM). Baseline (BL). *n*/*N* = 11/4. (**I**) Dopamine inhibition of NMDA EPSCs evoked from the mPFC in the presence (*n*/*N* = 13/5), but not absence (*n*/*N* = 6/2), of TBOA (20 μM). Data traces (*n/N* = 3/6) from non-TBOA–treated cells are taken from (F), reshown in (I). Summary data are means ± SEM. n.s., *P* > 0.05; *P < 0.05; **P < 0.01; ***P < 0.001. Statistical data and tests are provided in table S1.

Previous work using glutamate uncaging at single spines found that D2Rs modulate Ca^2+^ influx but not the overall current through NMDARs (*33*). As we found robust D2R inhibition of NMDAR currents when applying NMDA via iontophoresis, which likely activates both synaptic and extrasynaptic pools of NMDARs, we sought to examine how D2Rs modulate synaptic NMDARs. We began by investigating two of the major excitatory inputs to the NAc, those arising from the medial prefrontal cortex (mPFC) and those from the midline thalamus (mThal) (*57*–*59*). These inputs differ in their synaptic targeting: thalamic inputs synapse at spines and dendritic shafts (*60*–*63*), while cortical inputs preferentially target spines (*25*). To selectively activate these inputs, as well as remove any potential effect of presynaptic D2Rs, we injected an AAV-encoding Cre recombinase and an AAV-encoding Cre-dependent channelrhodopsin-2 (ChR2) into the mPFC or mThal [including the paraventricular (PVT) and mediodorsal thalamus] of D2R-floxed mice that had been crossed with D1-tdTomato mice (*Drd2^loxp/loxp^*; D1-tdTomato^+/−^) (Fig. 5, A and B). This strategy allowed us to selectively activate inputs where presynaptic D2R had been knocked out, as confirmed by the lack of effect of dopamine on the amplitude or paired-pulse ratio of evoked AMPA EPSCs (fig. S3). We recorded optogenetically evoked NMDAR EPSCs from tdTomato^−^ putative D2-MSNs 3 weeks later (Fig. 5C). While bath perfusion of either dopamine (100 μM) or quinpirole (1 μM) reduced the amplitude of NMDA EPSCs in D2-MSNs from mThal inputs, neither had an effect on NMDA EPSCs evoked from the mPFC (Fig. 5, D and E). This lack of modulation of mPFC EPSCs occurred despite dopamine effectively inhibiting NMDA currents in cells when evoked by iontophoresis (Fig. 5F). The lack of modulation at mPFC synapses was unlikely due to synapse-specific differences in NMDAR composition, as the kinetics of EPSCs evoked from the two inputs were similar and the GluN2B antagonist, ifenprodil, equally inhibited NMDA currents at mThal and mPFC inputs (Fig. 5G). Thus, D2R modulation of NMDARs is input specific, occurring at thalamoaccumbal but not corticoaccumbal synapses.

As corticoaccumbal synapses target the spine heads of D2-MSNs (*25*), whereas D2Rs are primarily located on dendrites in these cells (*25*, *27*), we next examined whether the lack of modulation at mPFC inputs was the result of D2Rs not being present at mPFC synapses. To test this, we examined whether increasing glutamate spillover would be sufficient to allow for recruitment of more distal extrasynaptic NMDARs at sites where D2Rs may be present. Blocking glutamate uptake with the excitatory amino acid transport blocker, DL-threo-β-benzyloxyaspartic acid (TBOA) (20 μM), increased the amplitude and the decay time of EPSCs evoked from mPFC inputs (Fig. 5H), consistent with enhanced glutamate spillover (*64*, *65*). In the presence of TBOA, subsequent application of dopamine was able to inhibit NMDA currents when evoked from mPFC inputs (Fig. 5I). Thus, D2Rs modulate NMDARs when evoked from the mPFC but only under conditions when spillover of glutamate is enhanced. Together, these results indicate that by restricting the spread of glutamate, transporters can limit the synapse-specific modulation of NMDARs by D2Rs.

### Dopamine acts at D2R-NMDAR heteromers to regulate striatal synaptic plasticity

**Fig. 6.**
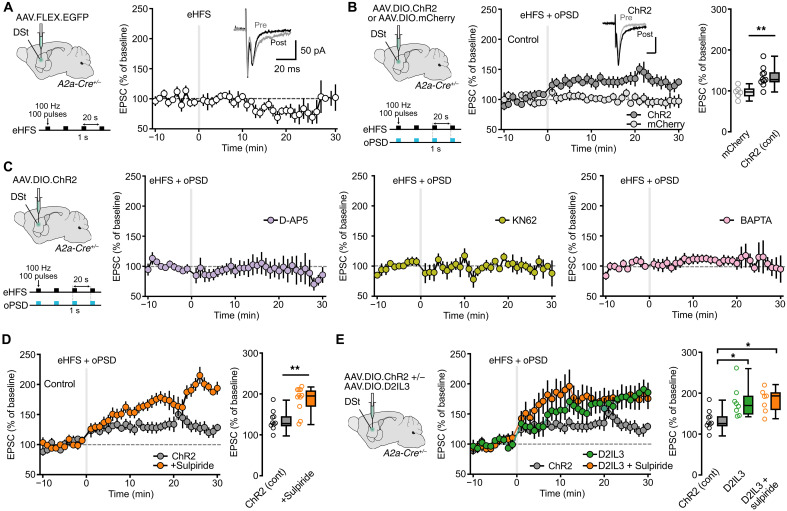
D2R-GluN2B cross-talk regulates striatal synaptic plasticity. (**A**) Striatal injection of AAV.FLEX.EGFP in A2a-Cre mice and schematic of eHFS protocol (four bursts of 100 stimuli at 100 Hz at an interval of 20 s). Quantification of electrically evoked EPSCs in dorsomedial striatum EGFP^+^ D2-MSNs (percentage of baseline) before and after the eHFS; *n*/*N* = 5/4. (**B**) Striatal injection of AAV.DIO.ChR2.mCherry (*n*/*N* = 10/9) or AAV.DIO.mCherry (*n*/*N* = 6/3) in A2a-Cre mice and schematic of eHFS paired with oPSD. Quantification of EPSC amplitude following eHFS + oPSD induction of LTP in ChR2^+^ D2-MSNs. (**C**) Block of LTP induction in ChR2^+^ D2-MSNs by D-AP5 (50 μM; *n*/*N* = 7/4), KN-62 (3 μM; *n*/*N* = 5/3), or intracellular dialysis of BAPTA (10 mM; *n*/*N* = 8/4). (**D**) Potentiation of LTP in ChR2^+^ D2-MSNs by sulpiride (1 μM; *n*/*N* = 11/9). Control (ChR2) data from (B) are reshown for comparison. (**E**) Striatal injection of AAV.DIO.ChR2.mCherry with or without injection of AAV.DIO.D2IL3 in A2a-Cre mice. The effect of D2IL3 expression on potentiation of LTP and lack of further effect by sulpiride (1 μM) were quantified. Control (ChR2) data from (B) are reshown for comparison. D2IL3, *n*/*N* = 8/5; D2IL3 + sulpiride, *n*/*N* = 7/4. Summary data are means ± SEM. *P < 0.05; **P < 0.01. Extended statistical data and tests are provided in table S1.

D2Rs play a critical role in regulating striatal synaptic plasticity including the induction of LTP (*66*–*72*). As GluN2B-containing NMDARs are key elements for the induction of LTP (*70*, *73*–*77*), we next examined the potential role that direct D2R modulation of NMDARs plays in gating striatal LTP. To isolate LTP mechanisms from endocannabinoid-mediated long-term depression (*78*–*80*), we included the cannabinoid receptor type 1 (CB1) antagonist AM251 (1 μM), while recording electrically evoked glutamatergic EPSCs in standard, Mg^2+^-containing aCSF. Consistent with previous work (*81*), we found that electrical high-frequency stimulation (eHFS) in the striatum was insufficient to induce LTP of EPSCs in D2-MSNs (Fig. 6A). However, following expression of ChR2 in D2-MSNs, pairing eHFS with optogenetically induced postsynaptic depolarization (oPSD) reliably triggered LTP in these cells (Fig. 6B) (*81*). The induction of LTP was prevented by the NMDAR antagonist D-AP5 (50 μM), the calmodulin-dependent protein kinase II (CaMKII) inhibitor KN-62 (3 μM), or chelating postsynaptic Ca^2+^ by including 1,2-bis(2-aminophenoxy)ethane-*N*,*N*,*N*′,*N*′-tetraacetic acid (BAPTA) (10 mM) in the patch pipette (Fig. 6C), confirming that LTP in D2-MSNs with this protocol is driven by postsynaptic NMDAR activation, Ca^2+^ influx, and CaMKII activation.

Application of the D2R antagonist sulpiride increased the magnitude of LTP, indicating that D2R activation by dopamine release evoked by eHFS opposes LTP (Fig. 6D and fig. S4). As D2Rs are widely expressed across circuits in the NAc, it is unclear whether this resulted from postsynaptic modulation of NMDAR or a presynaptic alteration of glutamate release. To elucidate the site of action and to directly test the role of D2R-NMDAR heteromers, we assessed LTP following the expression of the D2R IL3 minigene in D2-MSNs. Three weeks following minigene expression, the extent of LTP was enhanced in these cells (Fig. 6E and fig. S4). Notably, the increase in LTP in D2-MSNs expressing the D2R IL3 minigene was similar to that seen in control D2-MSNs in the presence of sulpiride (Fig. 6, D and E) and was not further potentiated by its subsequent addition (Fig. 6E and fig. S4). Collectively, these findings suggest that D2R activation exerts a brake on LTP by inhibiting NMDARs, likely through a postsynaptic interaction, and that disrupting this interaction relieves this inhibitory effect.

### D2Rs in the striatum are required for locomotion, motor learning, and active avoidance learning

**Fig. 7.**
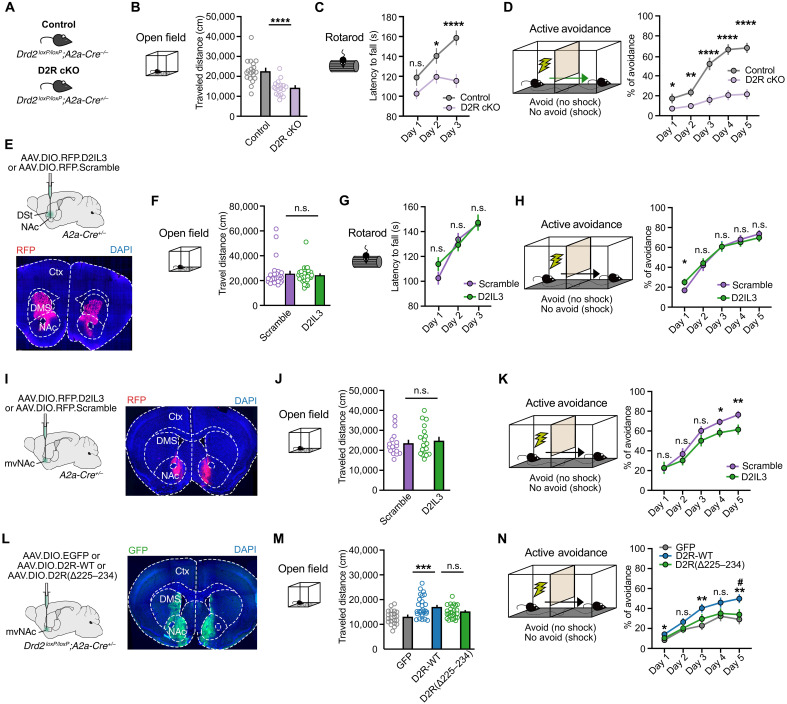
mvNAc D2R-GluN2B cross-talk regulates active avoidance learning. (**A**) Control and D2R cKO mice used for behavioral analysis in (B) to (D). (**B**) Open field (OF) test. Control, *N* = 19; D2R cKO, *N* = 18. (**C**) Accelerating RR test (3 days). Control, *N* = 19; D2R cKO, *N* = 18. (**D**) Active avoidance learning (5 days). Control, *N* = 22; D2R cKO, *N* = 21. (**E**) A2a-Cre mice expressing D2-IL3 minigene or scramble broadly in NAc and dorsal striatum (DMS), and resulting red fluorescent protein (RFP) expression [used for behavioral analysis in (F) to (H)]. (**F**) OF test in mice expressing D2-IL3 minigene (*N* = 32) or scramble (*N* = 27). (**G**) Accelerating RR test (3 days) in mice expressing D2-IL3 minigene (*N* = 32) or scramble (*N* = 26). (**H**) Active avoidance learning (5 days) of mice expressing D2-IL3 minigene (*N* = 32) or scramble (*N* = 26). (**I**) A2a-Cre mice expressing D2-IL3 minigene or scramble selectively in medial ventral NAc (mvNAc) and resulting RFP expression [used for behavioral analysis in (J) and (K)]. (**J**) OF test in mice expressing D2-IL3 minigene (*N* = 18) or scramble (*N* = 17). (**K**) Active avoidance (5 days) in mice expressing D2-IL3 minigene (*N* = 18) or scramble (*N* = 17). (**L**) D2R cKO mice expressing GFP, D2R-WT, or D2R(Δ225 to 234) selectively in mvNAc and resulting GFP expression [used for behavioral analysis in (M) and (N)]. (**M**) OF test in mice expressing GFP (*N* = 22), D2R-WT (*N* = 28), or D2R(Δ225 to 234) (*N* = 26). (*N*) Active avoidance learning (5 days) in mice expressing GFP (*N* = 23), D2R-WT (*N* = 29), or D2R(Δ225 to 234) (*N* = 27). #*P* < 0.05 D2R-WT versus D2R(Δ225 to 234). Summary data are means ± SEM. n.s., *P* > 0.05; *P < 0.05; **P < 0.01; ***P < 0.001; ****P < 0.0001. Extended statistical data, tests, and sex of animals are provided in table S1.

To determine how D2R-NMDAR heteromers modulate behavior, we examined several behaviors that are known to be regulated by D2Rs. Loss of D2R selectively in indirect pathway neurons (*Drd2*^*loxp/loxp*^; A2a-Cre^+/−^; D2R cKO) (Fig. 7A) resulted in reduced locomotor activity and motor coordination learning (Fig. 7, B and C, and fig. S5), consistent with previous findings (*82*). Global knockout of the D2 long-receptor isoform has been shown previously to severely impair active avoidance learning (*83*), an effect also seen with D2R antagonists (*84*), but it has not been clear whether this effect is related to loss or block of postsynaptic D2Rs in D2-MSNs as opposed to effects of D2R in other neurons. Therefore, we examined the performance of D2R cKO mice in shuttle box active avoidance (SAA), a type of aversive learning involving both associative and instrumental learning (*85*, *86*). Both male and female D2R cKO mice demonstrated profound deficits in avoidance learning across all days when compared with controls (*Drd2*^*loxp/loxp*^; A2a-Cre^−/−^), although both groups exhibited learning curves that plateaued by day 5 (Fig. 7D and fig. S5). These results demonstrate that postsynaptic D2Rs in striatal D2-MSNs are required for acquisition of active avoidance, as well as for locomotion and motor learning.

### D2R-NMDAR heteromers in the medial ventral NAc plays a role in active avoidance

Next, to examine whether D2R-NMDAR heteromerization is required for these behaviors, we virally expressed the D2R IL3 minigene in D2-MSNs in the dorsal and ventral striatum with injections that broadly covered these areas yet avoided the medial ventral NAc (mvNAc) (Fig. 7E). Mice expressing the D2R IL3 minigene and the scramble negative control had comparable locomotion in the open field (OF) (Fig. 7F and fig. S5) and the accelerating rotarod (RR) task (Fig. 7G and fig. S5), as well as normal avoidance learning (Fig. 7H and fig. S5), suggesting that while D2R is essential, direct interaction between D2Rs and NMDARs in the NAc core and dorsomedial striatum is not necessary for these behaviors.

Previous studies, however, have shown dopamine transmission to be consistently increased in the mvNAc when mice experienced aversive stimulation, with the response being much more variable in other parts of the ventral striatum (*87*, *88*), suggesting that the mvNAc may be a critical area for dopamine signaling in aversive learning. As the mvNAc was largely excluded in the minigene experiment described above, we next investigated whether disrupting the D2R-NMDAR interaction in mvNAc would disrupt active avoidance learning. Viral expression of the D2R IL3 minigene in D2-MSNs of the mvNAc and medial DStr (Fig. 7I) had no effect on locomotion in the OF, when compared to the scramble control (Fig. 7J and fig. S5), yet reduced avoidance performance at the end of training (Fig. 7K and fig. S5), suggesting that D2R-NMDAR heteromers in the mvNAc play a role in active avoidance.

To investigate whether restoration of D2R restricted to the mvNAc can rescue locomotor activity and active avoidance learning, we virally expressed Cre-dependent D2R-WT, D2R(Δ225 to 234), or GFP in the mvNAc of D2R cKO mice. Immunofluorescence staining showed that viral expression was primarily in the mvNAc and a limited area of the most rostral medial DStr (Fig. 7L). Although expression of D2R-WT in the mvNAc of D2R cKO mice increased locomotion compared to GFP, there was no significant difference between the D2R-WT– and D2R(Δ225 to 234)–rescued groups (Fig. 7M and fig. S5). Notably, D2R-WT rescue, but not D2R(Δ225 to 234), significantly improved active avoidance performance (Fig. 7N and fig. S5). Thus, expression of D2R-WT, but not the NMDAR interaction impaired mutant, in the mvNAc, improved active avoidance in D2R cKO mice. As we found that the behavioral effects of disrupting D2R-NMDAR interactions were consistent across sexes, with no significant sex × genotype interactions in the shuttle avoidance task or other key behavioral measures (fig. S5), data were pooled across sexes (Fig. 7). Together, the rescue and minigene heteromer disruption experiments establish that D2R-NMDAR cross-talk regulates active avoidance.

## DISCUSSION

This work reveals that D2Rs modulate NMDA currents in D2-MSNs through a physical coupling, independent of G protein and arrestin signaling. This modulation is input specific—occurring at thalamic but not cortical synapses—thereby resolving a nearly two-decade discrepancy regarding D2R effects on synaptic NMDARs. The cross-talk regulates synaptic plasticity and, within the medial ventral NAc, controls avoidance learning.

### Intracellular mechanisms of NMDA current modulation

MSNs are quiescent cells that require glutamate input to drive action potential firing (*89*). Hence, the strength of the input, as well as postsynaptic regulation of this input, determines MSN activity, ultimately determining what information is passed through the circuit. Dopamine importantly modulates both synaptic glutamatergic connections to the striatum and signaling within striatal MSNs in a cell type–specific manner (*72*, *90*). In D1-MSNs, convergent dopamine and glutamate signaling initiate a complex intracellular signaling cascade by which D1R activation potentiates NMDAR current through both canonical activation of cAMP/PKA signaling pathways (*91*) and cAMP-independent mechanisms involving Ca^2+^ activation of extracellular signal–regulated kinase signaling (*92*, *93*), as well as interactions mediated by heteromerization between these receptors (*36*, *92*, *94*, *95*). In D2-MSNs, the intracellular mechanisms regulating D2R modulation of NMDAR currents have been less clear. In dissociated striatal MSNs, activation of D2Rs was shown to reduce NMDA currents (*35*), whereas studies in striatal acute slice have demonstrated that although PKA phosphorylation decreases glutamate-evoked NMDAR Ca^2+^ permeability, dopamine itself does not alter NMDA current amplitude (*33*). Here, we found that D2R activation rapidly reduces NMDAR currents through a process independent of PKA, cAMP signaling, G protein, and arrestin signaling.

In addition to modulating NMDARs through intracellular signaling cascades, D2Rs can engage in direct physical interactions with NMDARs to modulate their activity. Evidence from coimmunoprecipitation within PSD fractions derived from rat hippocampus, prefrontal cortex, and striatum, as well as proximity ligation assay (PLA), revealed a direct association between NMDARs at the GluN2B subunit and D2Rs at the IL3, region T225-A234 (*35*, *36*). In agreement with these data, we found that D2R-mediated inhibition of NMDAR currents was eliminated by blocking D2-NMDAR interactions, either by using a Cre-dependent minigene encoding this D2R-IL3 sequence or through expression of a truncated D2R variant lacking the T225-A234 residues.

By physically coupling to NMDARs, D2Rs enable rapid integration of dopaminergic and glutamatergic signals, bypassing the temporal constraints of second messenger–based regulation. Supporting this, our dual iontophoresis study revealed that dopamine can inhibit NMDAR-mediated currents within ~150 ms. Although a peptide from D2R IL3 and a recombinant NR2B C-tail fragment directly interacted (*35*), we cannot rule out a role for other proteins in the cellular context. Dendritic NMDARs are integrated into a large macromolecular signaling complex organized by scaffold and adaptor proteins, linking them to kinases, phosphatases, and other downstream signaling molecules (*96*), and we cannot exclude the possibility that the D2R-NMDAR complex is part of a larger modulatory complex that regulates this activity. Future work will be needed to decipher the biophysical mechanism by which D2Rs alter NMDAR activity, potentially through modulation of channel gating or ion permeability.

### Modulation of synaptic NMDARs

While work in cultured neurons applying NMDA exogenously has observed D2R-mediated reduction in NMDAR currents (*35*), this modulation has not previously been observed at glutamatergic synapses. An initial study reported minimal changes in striatal NMDAR currents following D2R activation in response to electrical stimulation of neocortical afferents (*97*). Similarly, striatal NMDAR currents in dendritic spines evoked by two-photon glutamate uncaging were unaffected by D2R agonists (*33*). Our finding that D2Rs selectively modulate thalamic but not cortical inputs directly resolves this discrepancy—glutamate uncaging at spine heads would activate NMDARs that are not in proximity to dendritic D2Rs. We found that dopamine does not modulate optogenetically evoked NMDA EPSCs from mPFC inputs unless glutamate spillover is enhanced. As cortical inputs preferentially form synapses on dendritic spines (*25*, *98*), while D2Rs are primarily located perisynaptically (*25*, *27*), the absence of modulation at cortical inputs likely results from a lack of D2Rs at spine synapses.

In addition to the corticostriatal pathway, the striatum also receives dense innervation from the thalamus, hippocampus, and amygdala, each with distinct synaptic properties (*5*, *59*, *99*). Thalamic inputs have been found to terminate at spine heads and dendritic shafts (*60*–*63*, *100*, *101*). Since we found that D2Rs modulate thalamic NMDA EPSCs, we hypothesize that D2R-NMDAR interactions occur at dendritic shaft synapses contacted by thalamic inputs. As shaft synapses are thought to be more stable than spine synapses, which are often dynamic with frequent reorganizing of nanodomains and surface receptors (*102*), D2R-NMDA heteromers may serve to stabilize receptor surface localization, limit lateral mobility, and regulate synaptic receptor distribution. By selectively inhibiting thalamic NMDA EPSCs, D2Rs may be acting to reduce their effectiveness at driving D2-MSNs to action potential threshold, thereby diminishing “noise” from these inputs and allowing the striatum to prioritize more relevant stimuli from regions such as the mPFC, where modulation is only seen when synaptic spillover of glutamate is induced. Thus, dopamine may function as an input selector, acting to determine which inputs convey information to striatal MSNs. While cortical and thalamic inputs both converge onto D2-MSNs, we speculate that rather than broadly modulating all excitatory inputs, dopamine instead functions via heteromeric D2R-NMDAR interactions to filter the strength of inputs to allow for input-selective neuromodulation.

### D2R modulation of NMDA current in LTP and implications in addiction and pathological dopamine-driven plasticity

Accumulating evidence highlights the critical role of synaptic plasticity in the striatum for motor control and learning, with dopaminergic inputs serving as a key regulator (*103*, *104*). Striatal NMDARs induce LTP of AMPAR-mediated transmission, contributing to memory and learning processes (*72*). Mechanistically, back-propagating action potentials (bAPs) facilitate removal of the Mg^2+^ block from NMDARs, therefore allowing Ca^2+^ influx into postsynaptic neurons (*105*). The location of D2Rs on dendrites places them in an ideal location to regulate bAP, where they can function to reduce NMDAR currents, potentially supporting a mechanism by which D2Rs modulate corticostriatal LTP. Supporting this, we found that disrupting the interaction between D2R and NMDAR facilitates CaMKII-dependent LTP induction. In this scenario, the GluN2B subunit, now free of D2R inhibition, may promote enhanced Ca^2+^ influx and enable CaMKII binding to GluN2B (*106*–*108*), facilitating synaptic plasticity. The GluN2 subunit of NMDARs largely determines receptor properties, with GluN2B having slower decay kinetics and a higher calcium permeability than GluN2A (*109*). Therefore, by selectively inhibiting GluN2B-NMDARs, D2Rs may be functioning to attenuate prolonged calcium influx, effectively diminishing the sustained signaling and plasticity associated with GluN2B activity. The role of D2R-NMDAR interactions in gating NMDAR calcium transients, as well as the impact that this has on regulating plasticity at accumbal projections, remain intriguing unanswered questions. Cocaine administration blocks dopamine reuptake transporters, creating an increased dopamine “tone,” or hyperdopaminergic state that disrupts dopamine’s fine-tuning of glutamatergic signaling. In vivo cocaine exposure also increases the proportion of D2Rs that form heteromers with NMDARs in the NAc (*36*), suggesting a possible mechanism by which NMDAR current modulation may also be increased. To explore the relationship between dopaminergic hyperfunction and NMDAR current, we used both in vivo cocaine exposure and homozygous DAT-Cre mice to promote increased dopamine tone. As predicted, we found that this leads to enhanced NMDA current modulation in both models. We interpret the enhanced modulation as most likely reflecting increased D2R-NMDAR heteromer formation, as supported by the PLA data from Andrianarivelo *et al.* (*36*) following chronic cocaine. Although overall D2R levels were reported to be unchanged in DAT-Cre mice by ligand binding autoradiography (*110*), we cannot fully exclude a contribution from changes in local D2R density at relevant dendritic compartments. Considering that D2Rs suppress NMDA-mediated current and calcium influx, this would indicate that hyperdopaminergic states, by increasing the modulatory effect of D2Rs, would also be expected to further suppress striatal plasticity. We found that LTP induction was completely abolished in homozygous DAT-Cre mice, an effect that could be reversed by either application of the D2R antagonist sulpiride or expression of the minigene that blocks D2R-NMDAR heterodimerization (fig. S4). Given that cocaine exposure promotes the redistribution of NMDARs from synaptic to extrasynaptic sites (*111*), these findings suggest that cocaine exposure promotes a shift toward extrasynaptic signaling by up-regulating and redistributing GluN2B-containing NMDARs while increasing D2R-GluN2B heteromers. This shift likely disrupts the balance between synaptic and extrasynaptic signaling, favoring maladaptive processes over neuroplastic responses. Cocaine administration induces a range of structural and functional adaptations at glutamatergic and dopaminergic synapses in the NAc, including changes in spine density, synaptic strength, and dopaminergic connectivity, although these adaptations are highly regimen-dependent and their causal relationships remain complex (*112*–*114*). It will be interesting for future work to examine how D2R modulation of NMDA currents affects LTP and long-term depression (LTD) induction at various glutamatergic inputs to the NAc and the projection-specific adaptations that occur in these circuits following long-term psychostimulant exposure.

The increased dopamine tone observed in the striatum of patients with schizophrenia, detectable as early as the prodromal phase (*115*, *116*), would also be expected to increase D2R-NMDAR heteromer formation. This becomes particularly notable, given that *GRIN2A* genetic variants confer substantial risk for schizophrenia (*117*–*119*). GluN2A knockout in mice is sufficient to evoke behavioral changes associated with the disease, and differential depletion of GluN2A induces heterogeneous schizophrenia-related phenotypes in mice (*119*). While speculative, GluN2A loss would be expected to lead to a substantial increase in D2R-GluN2B heterodimers, fundamentally altering cross-talk dynamics. This framework suggests that antipsychotic drugs, which block D2R-NMDAR cross-talk, may exert their therapeutic effects, at least in part, by disrupting this pathological cross-talk rather than solely through canonical D2R antagonism. If this were the case, then therapeutics directed at the heteromer more directly may ultimately have promise for improved treatment with reduced side effects.

While our direct measurements of NMDAR currents relied on exogenous dopamine application, our LTP experiments demonstrate that dopamine released in response to electrical stimulation engages the heteromer. Whether tonic dopamine is sufficient to constitutively engage this mechanism under basal conditions remains an important open question for future investigation.

### A role for D2R-NMDAR heteromers in active avoidance learning

SAA, a paradigm used to study aversive learning, comprises two major phases: fear conditioning (cue-shock association) and instrumental learning (shock avoidance) (*85*, *86*). Previous work showed that MSNs in different striatal regions receive regional and temporally dynamic glutamate and dopamine inputs from cortex, thalamus, and VTA during aversive learning. In particular, posterior D2R^+^ PVT glutamate signaling decreases during fear conditioning in the NAc but then increases during avoidance and is necessary for successful avoidance learning (*120*). More broadly, PVT-NAc projections have been shown to mediate aversive behaviors and active avoidance across multiple contexts (*121*, *122*). Dopamine transmission in the mvNAc is enhanced by unexpected aversive outcomes and is required for initial cue-shock association, while dopamine transmission in the NAc core responds more dynamically to aversive stimulation, strengthening across learning to encode prediction errors and guide consolidation of avoidance behaviors (*87*, *123*). At the cellular level, circuit studies have shown that D1-MSNs signal salience, while D2-MSNs respond to prediction errors (*124*). However, the molecular mechanisms by which dopamine modulates glutamatergic signaling at D2-MSNs during aversive learning remain poorly understood.

Previous pharmacological and genetic studies have implicated D2Rs in the NAc in aversive learning (*83*, *84*), but the specific contribution of postsynaptic D2Rs, particularly in distinct NAc subregions, remained unclear. We found here that loss of postsynaptic D2Rs in D2-MSNs results in severe deficits in avoidance learning (as well as in motor learning and locomotion). Furthermore, we found that D2R-NMDAR interaction in D2-MSNs in the mvNAc is required for normal performance in the active avoidance task, as interfering with D2R-NMDAR interactions specifically in the mvNAc reduced performance. In addition, WT D2Rs, but not the mutant D2R(Δ225 to 234), expressed only in the mvNAc partially rescued avoidance learning in D2R cKO mice. Notably, loss of D2R-NMDAR interactions produced a less severe phenotype than knockout of D2R, suggesting that D2R-NMDAR interaction in the mvNAc is not the only mechanism at play in active avoidance learning. D2R signaling through other downstream pathways in other striatal areas may also play a role, although we cannot rule out a developmental effect in knockout animals that cannot be fully rescued in adulthood.

The requirement for D2R-NMDAR in mvNAc occurs within this complex landscape of glutamate and dopamine dynamics, although the precise relationship remains unclear. While PVT-NAc glutamate signal decreases during fear conditioning and increases during avoidance execution, D2R-NMDAR cross-talk would modulate postsynaptic NMDAR responses based on dopamine release and may play a role during integration of multiple glutamate inputs beyond PVT, as well as fine-tuning NMDAR sensitivity to released glutamate. Further work is needed to determine whether D2R-GluN2B cross-talk is most important during fear conditioning, avoidance execution, or transitions between these phases.

An apparent paradox of our findings is that D2R-NMDAR heteromers suppress NMDAR-dependent LTP, yet disrupting heteromers in mvNAc impairs threat-avoidance learning rather than enhancing it. This is consistent with established findings that unrestrained synaptic potentiation can impair, rather than facilitate, learning, either through saturation of plasticity mechanisms or loss of the bidirectional flexibility required for associative encoding (*125*–*127*). We propose that heteromers function as input-selective gates that constrain potentiation at specific synapses, preserving the dynamic range necessary for behaviorally relevant plasticity. The precise circuit logic linking input-specific heteromer function to avoidance behavior, including whether this reflects biased engagement of D1-MSN pathways, tuned NMDAR gain during learning, or preservation of bidirectional plasticity at PVT → NAc synapses, remains an important question for future investigation.

Together, these findings extend our previous work showing that distinct D2R signaling pathways can control different behaviors: Arrestin signaling mediates locomotion, whereas G protein signaling controls incentive motivation (*46*). Here, we show that D2R-NMDAR interaction controls avoidance learning. This emerging paradigm—where different D2R downstream mechanisms link to specific behaviors—suggests the possibility of more precise therapeutic targeting. Rather than broadly blocking D2R, selective modulation of arrestin signaling, G protein signaling, or D2R-NMDAR heteromers could potentially treat distinct pathophysiological symptoms without disrupting normal functions. Future work monitoring real-time glutamate and dopamine signals during avoidance learning will clarify how this molecular mechanism contributes to different learning phases and its therapeutic specificity.

## MATERIALS AND METHODS

### Animals

All animal breeding, housing, and experimental procedures were performed according to protocols approved by Institutional Animal Care and Use Committee at the University of Colorado School of Medicine (0155) and the New York State Psychiatric Institute (NYSPI-1706-T). Mice of both sexes were used for experiments. Ambient housing temperature was maintained at ~25.5°C with 40 to 60% humidity. Food and water were available ad libitum, and experiments were conducted during the light phase. Behavioral and electrophysiology experiments were conducted on *Adora2a*-Cre [Mutant Mouse Resource and Research Center (MMRRC), 036158-UCD], *Drd1*-Cre (MMRRC, 034258), D1-tdTomato (the Jackson Laboratory, 016204), *Drd2*-floxed (the Jackson Laboratory, 020631), and DAT–internal ribosomal entry site (IRES)–Cre (the Jackson Laboratory, 006660) mice. Lines crossed with these animals were bred in the laboratory.

### Stereotaxic injections

For animals used in subsequent electrophysiology studies, mice (3 to 6 weeks) were anesthetized with isoflurane, transferred to a stereotaxic apparatus (Kopf Instruments), and kept under constant 2% isoflurane anesthesia. The skull surface was revealed via a midline sagittal incision. All AAV viruses were bilaterally injected into the dorsal and ventral part of the striatum using a Nanoject III (Drummond Scientific) at 100 nl/min. Mice were then allowed to recover for 3.5 to 6 weeks to allow for viral expression. Coordinates in millimeters from bregma were as follows: DStr [anterior-posterior (AP), +1.2; medial-lateral (ML), ±1.85; dorsal-ventral (DV), −3.3], NAc (AP, +1.5; ML, ±1.15; DV, −4.3), GPe (AP, −0.35; ML, ±1.3; DV, −3.5), mPFC (AP, +2.1; ML, ±0.4; DV, −1.5), mThal (AP, −1.2; ML, ±0.4; DV, −3.4). The pipette was kept at the site for 5 min and then slowly withdrawn. For fluorescent labeling of neurons, 300 nl of AAV5.EF1a.DIO.EYFP (Addgene, 27056), AAV9.CAG.FLEX.EGFP (Addgene, 50465), or AAV5.CAG.tdTomato (Addgene, 59462) were used. GIRK2 was overexpressed by injecting AAV9.hSyn.DIO.tdTomato.T2A.GIRK2 (300 nl; University of Pennsylvania Viral Core, V5688) into A2a-Cre or D2R cKO mice. D2R-WT, D2R-ARB, and D2R(Δ225 to 234) were expressed in D2-MSNs by injecting AAV9.EF1a.DIO.D2L.P2A.eGFP (200 nl), AAV9.EF1a.DIO.D2R-ARB.P2A.eGFP (200 nl), or AAV9.EF1a.DIO.D2R(Δ225 to 234).P2A.eGFP (200 nl) into A2a-Cre or D2R cKO mice either on their own or in combination with AAV9.hSynapsin.DIO.tdTomato.T2A.GIRK2 (300 nl). D2R-WT was expressed D1-MSNs by injecting AAV9.EF1a.DIO.D2L.P2A.eGFP (200 nl) with or without AAV9.hSynapsin.DIO.tdTomato.T2A.GIRK2 (300 nl) into D1-cre mice. hM4Di was expressed in D2-MSNs by injecting AAV5.hSyn.DIO.hM4D(G_i_).mCherry (500 nl; Addgene, 44362) into the A2a-Cre mice. D2R IL3 or scramble control were expressed by injecting AAV9.DIO.EF1a.RFP.P2A.D2IL3 (500 nl; Virovek) or AAV9.DIO.EF1a.RFP.P2A.scramble (500 nl; Virovek) into A2a-Cre mice either on their own or in combination with AAV9.hSyn.DIO.tdTomato.T2A.GIRK2 (300 nl) or AAV5.EF1a.DIO.EGFP (300 nl). For CRISPR-SaCas9–mediated arrestin knockout studies, AAV9.FLEX.SaCas9.U6.sg.sg135 (300 nl) or AAV9.FLEX.SaCas9.U6.sg.sg*Rosa* (300 nl) were injected into A2a-Cre mice with either AAV9.hSyn.DIO.tdTomato.T2A.GIRK2 (300 nl) or AAV5.EF1a.DIO.EGFP (300 nl). For NMDA knockout studies, AAV5.EF1a.DIO.mCherry (300 nl) with or without AAV5.FLEX.SaCas9.U6.sgGrin1 (300 nl) was injected into A2a-Cre mice. For optogenetic excitation of mThal and mPFC inputs, *Drd2^loxp/loxp^*; D1-tdTomato^+/−^ mice were injected with pAAV5.EF1.dflox.hChR2(H134R)-mCherry.WPRE.hGH (200 nl; Addgene, 20297-AAV5) and AAV5-Ef1a-mCh-Cre (200 nl; UNC Vector Core, AV6144B). For LTP experiments, A2a-Cre mice were injected with 300 nl of AAV5.EF1a.DIO.hChR2(H134R)-EYFP (Addgene, 20298) or AAV5.CAG.tdTomato. For examination of effect of minigene for LTP experiments, 300 nl of AAV5.EF1a.DIO.hChR2(H134R)-EYFP (Addgene, 20298) was injected into A2a-Cre mice with 200 nl of AAV9.DIO.EF1a.RFP.P2A.D2NR2b or AAV9.DIO.EF1a.RFP.P2A.scramble. For retrograde labeling, AAVrg.hSyn.Cre.WPRE.hGH (150 nl; Addgene, 105553) was injected into the GPe, and AAV9.CAG.FLEX.EGFP (300 nl; Addgene, 50465) was injected into the DStr. For examination of minigene effect on DAT^IREScCre/IREScre^, 150 nl of AAVrg.hSyn.Cre.WPRE.hGH was injected into the GPe, and 300 nl of AAV9.CAG.FLEX.EGFP (Addgene, 50465) was injected into the DStr with or without AAV9.DIO.EF1a.RFP.P2A.D2NR2b.

For animals used in subsequent behavioral studies, AAV stereotaxic injections were performed on mice aged 2 to 8 months, as described above with minor modifications. Anesthesia was induced with an intraperitoneal injection of ketamine (80 mg/kg) and xylazine (5.6 mg/kg) and maintained with isoflurane (0.3 to 1% in room air) delivered at 30 to 50 ml/min using an isoflurane vaporizer (SomnoSuite). AAV stocks were diluted in phosphate-buffered saline (PBS) containing 0.001% Pluronic F-68 to equalize concentrations between control and experimental viruses: 1.3 × 10^13^ vg/ml for D2R and truncated mutant AAVs and 2 × 10^13^ vg/ml for D2R IL3 and scrambled control AAVs. AAVs were bilaterally injected into the striatum using a Nanoject II (Drummond Scientific) at a rate of 50 nl/min. Injection coordinates (in millimeters from bregma) were as follows: for expressing minigene in the dorsal and ventral striatum, AP of +1.5, ML of ±1.25, and DV of −4.1 and −2.9; for expressing minigene in the mvNAc, AP of +1.54, ML of ±0.5, and DV of −4.5; and for expressing WT and mutant D2 in the mvNAc, AP of +1.5, ML of ±0.9, and DV of −4.8. A total volume of 350 nl was injected for DStr, and 250 nl for ventral striatum or mvNAc. After injection, the pipette was held in place for 10 min before being slowly withdrawn. D2R IL3 or the scramble control was expressed by injecting AAV9.DIO.EF1a.RFP.P2A.D2IL3 or AAV9.DIO.EF1a.RFP.P2A.scramble into A2a-Cre mice. GFP, D2R-WT, and D2R(Δ225 to 234) were expressed by injecting AAV.EF1a.DIO.EGFP, AAV9.EF1a.DIO.D2L.P2A.eGFP, or AAV9.EF1a.DIO. D2R(Δ225 to 234).P2A.eGFP into D2R cKO mice.

### Acute brain slice preparation

Coronal brain slices containing the striatum/NAc were collected at 240 μm in thickness. Mice (6.5 to 12 weeks) were anesthetized with isoflurane and transcardially perfused with an ice-cold sucrose-based cutting solution (95% O_2_ and 5% CO_2_) containing 75 mM NaCl, 2.5 mM KCl, 6 mM MgCl_2_, 1.2 mM NaH_2_PO_4_, 25 mM NaHCO_3_, 0.1 mM CaCl_2_, 11.1 mM d-glucose, and 1 mM kynurenic acid. Brains were then extracted and sectioned using a vibratome (Leica VT1000 S, Leica Biosystems) in the cutting solution. Slices were transferred to an oxygenated chamber maintained at 34°C and perfused with Mg^2+^-free aCSF containing 126 mM NaCl, 2.5 mM KCl, 2.5 mM CaCl_2_, 1.2 mM NaH_2_PO_4_, 21.4 mM NaHCO_3_, and 11.1 mM d-glucose. After an incubation period of 45 to 60 min, slices were moved to a submerged recording chamber, where they were continuously perfused with aCSF at a flow rate of 2 ml/min. MSNs were visually identified using an Olympus BXWI51 microscope equipped with custom-built infrared gradient contrast optics. Fluorescence imaging was performed using light-emitting diode illumination (Thorlabs).

### Whole-cell slice electrophysiology

MSNs were voltage clamped in whole-cell configuration at −70 mV using Axopatch 200B amplifiers (Molecular Devices). Signals were acquired using Axograph X (Axograph Scientific) at a sampling rate of 5 kHz (filtered to 2 kHz) and LabChart (AD Instruments) at 1 kHz. Patch pipettes (1.5 to 2 MΩ; World Precision Instruments) were used for recording. For electrical stimulation, dopamine or glutamate release was evoked by applying a brief (0.5 ms) electrical pulse with a monopolar glass stimulating electrode filled with aCSF.

For recordings of NMDAR-mediated currents, picrotoxin (100 μM) and 6,7-dinitroquinoxaline-2,3-dione (DNQX; 10 μM) were added to the aCSF to inhibit GABA_A_ and AMPARs, and a Cs-based internal solution was used, which contained 135 mM CsMeSO_3_, 0.1 mM CaCl_2_, 2 mM MgCl_2_, 10 mM Hepes(K), 0.1 mM EGTA, adenosine triphosphate (ATP; 1 mg/ml), GTP (0.1 mg/ml), sodium phosphocreatine (1.5 mg/ml), and 10 mM QX-314, adjusted to pH 7.34 and 275 milliosmoles (mOsm). For recording AMPA:NMDA ratios, MSNs were held at −70 mV in aCSF lacking DNQX to measure AMPAR-mediated currents, and DNQX (10 μM) was then applied. Cells were then stepped to +40 mV to measure NMDAR-mediated currents. AMPA and NMDA traces were digitally subtracted in the presence of DNQX (10 μM) and AP5 (50 μM) to remove the stimulus artifact for clarity. For the data in Fig. 1, QX-314 was not included in the intracellular pipette as tetrodotoxin (200 nM) was used in the aCSF.

For recordings of D2-IPSCs, whole-cell recordings were made from tdTomato^+^ D2-MSNs or D1-MSNs when overexpressing D2Rs in D1-MSNs. The internal solution contained: 135 mM potassium gluconate, 0.1 mM CaCl_2_, 2 mM MgCl_2_, 10 mM Hepes(K), 10 mM BAPTA, ATP (1 mg/ml), GTP (0.1 mg/ml), and sodium phosphocreatine (1.5 mg/ml), adjusted to pH 7.4 and 275 mOsm. D2-IPSCs were isolated in presence of 1 μM SCH23390, 200 nM scopolamine, 300 nM CGP55845, 10 μM DNQX, and 1 μM dihydro-β-erythroidine (DHβE) and elicited once per minute.

NMDA was applied via iontophoresis using an iontophoretic electrode that was filled with 200 mM NMDA, and NMDA was injected with a negative current (−100 nA) and maintained with a retention current (+3 to +10 nA) to prevent leakage. Aliquots of NMDA (200 mM) were made by dissolving NMDA in 1 ml of NaOH (1 N) and 4 ml of water, which was sonicated and pH adjusted to 7.3 and then frozen until used. In a subset of neurons, cells were filled with Alexa Fluor 594 (5 μM) via the recording pipette to visualize dendrites. Iontophoresis pipettes were positioned close to dendrites away from the soma. Dopamine and quinpirole were bath applied via perfusion except in the case when dopamine was applied via iontophoresis, in which case an iontophoretic electrode was filled with 1 M dopamine, and dopamine was injected with a positive current (−50 nA) and maintained with a retention current (−1 to −5 nA) to prevent leakage. For examining mThal and mPFC inputs, recordings were performed in Mg^2+^-free aCSF containing picrotoxin (100 μM), 6-nitro-7-sulfamoylbenzo[*f*]quinoxaline-2,3-dione (NBQX; 100 μM), DhβE (1 μM), and AM251 (3 μM). Putative D2-MSNs were identified by the absence of tdTomato expression. Presynaptic glutamate release was stimulated via a 3-ms flash of blue light. Postsynaptic MSNs were held at −70 mV, and NMDAR EPSCs were evoked every 30 s. For experiments examining synaptic spillover, glycine (10 μM) was added to aCSF in addition to TBOA (20 μM). Modulation of NMDA currents was measured as the percentage change from baseline following D2R agonist application taken as the average of three to five consecutive responses. If no change was observed, then modulation was taken as the average of three to five responses, 3 min following bath perfusion of dopamine. Dopamine was consistently applied to slices for 5 min per recording. For LTP experiments, a monopolar glass stimulating electrode was positioned ~150 μm away from the recording electrode. The recordings were conducted in regular aCSF solution containing 1.2 MgCl_2_, with picrotoxin (100 μM) and AM251 (3 μM). Cs-based internals were used for recordings. AMPAR-EPSCs were evoked every 30 s. After establishing a stable baseline for 10 min, HFS was applied via the stimulating electrodes. HFS consisted of four trains of 100 pulses delivered at 100 Hz, with a 20-s interval between trains. For eHFS paired with oPSD (eHFS + oPSD), D2-MSNs were stimulated with 470-nm light through the objective lens (1-s duration each) to induce oPSD.

### Immunohistochemistry

Mice were anesthetized with isoflurane and perfused transcardially with ice-cold PBS containing 137 mM NaCl, 1.5 mM KH_2_PO_4_, 8 mM NaH_2_PO_4_, and 2.7 mM KCl (pH 7.4), followed by paraformaldehyde [4% (w/v) in PBS]. After extraction, brains were placed in 4% paraformaldehyde at 4°C for 16 to 24 hours for fixation and then transferred to 30% sucrose in PBS for at least 48 hours. Coronal brain sections (30 μm) were prepared using a microtome (Leica Microsystems, CM-1950). Sections were mounted using ProLong Gold with 4′,6-diamidino-2-phenylindole (DAPI; Invitrogen, P36930), imaged using a Zeiss LSM780 (Carl Zeiss) or a VS120 Slide Scanner (Olympus), and analyzed with ImageJ. GFP was detected without staining.

For histology following completion of behavioral studies, mice were anesthetized after undergoing behavioral assessment using ketamine and xylazine and perfused transcardially with PBS, followed by 4% paraformaldehyde in PBS. Brains were post-fixed at 4°C for 16 to 24 hours, and vibratome sections (50 μm) were prepared as described above, except that the GFP signal was amplified with a chicken anti–GFP-1020 (Aves Labs; 1:1000 dilution) and goat anti-chicken Alexa Fluor 488 immunoglobulin Y (H + L) (Thermo Fisher Scientific).

### HEK293T cell signaling assays

The pcDNA3.1 mammalian expression plasmid encoding the WT human dopamine D2R (long isoform) was described previously (*46*, *128*). The pcDNA3.1 plasmid encoding D2R(Δ225 to 234) was constructed using standard techniques in molecular biology. Briefly, the D2R-WT plasmid above was digested with Bst EII and Bsu 36I, removing a segment of the receptor-coding region that includes the putative GluN2B interacting motif (T225-A234). A synthetic double-stranded linear DNA fragment encoding the same removed region (Thermo Fisher Scientific), but with the codons for the T225-A234 motif deleted, was cloned into the Bst EII and Bsu 36I sites of the digested plasmid, and the resulting pcDNA3.1 D2R-trunc plasmid was confirmed by DNA sequencing (Psomagen).

All HEK cell–based assays were carried out in 293T cell [American Type Culture Collection (ATCC), CRL-3216] from *Homo sapiens* embryonic kidney tissue (HEK293T), which are female in origin and authenticated by the manufacture using short tandem repeat analysis to determine species and unique DNA profile. The HEK293T cells used in this study tested negative for mycoplasma contamination. These cells were maintained in Dulbecco’s modified Eagle’s medium, high glucose (Gibco), supplemented with 10% fetal bovine serum (Corning Inc.) and 1% penicillin-streptomycin (Corning Inc.) at 37°C with 5% CO_2_. HEK293T were grown to ~70% confluency in 10-cm dishes or six-well-plate tissue culture plates and were assayed 48 hours post–transient transfection (see below for details related to each specific assay). To measure D2R-mediated cAMP inhibition, we used a previously described bioluminescence resonance energy transfer (BRET)–based cAMP assay (*129*). For this assay, the D2R-WT or D2R(Δ225 to 234) plasmids (0.5 μg) and the plasmid encoding the cAMP sensor using YFP-Epac-RLuc (CAMYEL; 1 μg; ATCC) were transfected using lipofectamine 2000 for cAMP assay (Thermo Fisher Scientific) per the manufacturer’s protocol (*130*). To measure β-arrestin interactions with D2R, we used a bystander BRET assay that detects recruitment of β-arrestin2 to the plasma membrane upon dopamine stimulation of receptors (*130*, *131*). For this assay, HEK293T were grown to ~70% confluency in six-well-plate and transfected with a 1:2 ratio of DNA:polyethylenimine. The plasmids described above coding for D2R-WT or D2R-trunc (0.33 μg per well), *Renilla reniformis* luciferase 8–β-arrestin2 (0.042 μg per well), plasma membrane–anchored citrine (0.83 μg per well), and noncoding plasmid (pcDNA5 FRT; 1.29 μg per well) were transfected.

For both assays, the transfected cells were prepared in a 96-well microplate format and after incubation with coelenterazine H (5 μM; Dalton Pharma Services) for 8 min, dopamine hydrochloride (MilliporeSigma) or vehicle was injected, and the cells were assayed 10 min after agonist stimulation as described previously using a PHERAstar FS plate reader (BMG Labtech) (*130*). For the cAMP assays, 10 μM forskolin (Cayman Chemical Company) was added to the cells 10 min before dopamine injection. The BRET ratio calculation and subsequent data analysis were carried out as described previously (*46*, *130*).

### Behavior

For behavioral studies, male and female mice were group housed separately, and a plastic dome was provided as postsurgical cage enrichment. At least 4 weeks after surgery, mice (>3 months old) underwent OF test, accelerating RR, and SAA testing. In most cases, the same animals were tested sequentially in the OF, accelerating RR, and SAA tasks. For minigene expression in the mvNAc, the animals were only tested in OF and SAA tasks.

### Open field

The OF test was used to assess novelty-induced activity. Home cage changes were avoided within 24 hours before the test. Mice were weighed and tail marked the day before the test. On the test day, mice were transferred to testing room in home cage with food and water and acclimated for 60 min. Each mouse was then placed in the center of a 2 foot–by–2 foot (0.61 m–by–0.61 m) Plexiglas chamber for a 60-min session. Horizontal and vertical activities were recorded via infrared beam breaks lining the bottom of the chambers. After testing, mice were returned to their home cages.

### Accelerating RR

The accelerating RR test was used to evaluate motor coordination learning. Mice were transferred to the testing room in their home cages and acclimated for 30 min. Each mouse was placed on a cleaned and dried five-lane RR apparatus (Ugo Basile), rotating initially at 5 rotations per minute (RPM). Once all mice were facing forward, the trial began and the rotation speed increased linearly from 5 to 80 RPM over 5 min. Mice were removed from the apparatus if they fell or met specific criteria—such as spinning continuously for more than two full rotations or spinning three times during a trial—and returned to their home cages. Each mouse underwent three trials per day, with a maximum trial duration of 5 min and a 10-min intertrial interval, for 3 consecutive days.

### Shuttle box active avoidance

SAA training was conducted using shuttle boxes (MED Associates Inc.) equipped with eight infrared photobeams and metal grid floors capable of delivering foot shocks. The boxes were housed in sound-attenuated chambers with continuous fan-generated white noise. Shuttle doors remained open throughout all habituation and testing sessions, allowing free movement between the two chambers. Before training days, mice were habituated in the shuttle boxes for 30 min. On training days, each session began with a 10-min pretest habituation. Each trial consisted of a 7-s auditory tone (12 to 14 kHz, ~70 dB), followed, if the mouse did not run to the opposite chamber, by a 1-s, 0.25-mA foot shock delivered to the chamber where the mouse was located. If the mouse shuttled to the opposite chamber before the tone ended, then the shock was prevented (and the tone would turn off), and the trial was recorded as an avoidance. If the mouse crossed after the shock onset, then the trial was recorded as an escape. Each mouse completed 30 trials per day, with intertrial intervals randomly ranging from 10 to 30 s, for 5 consecutive days.

### Chemicals

Picrotoxin, MK-801, sulpiride, DNQX, NBQX, AP5, SCH23390, DhβE, scopolamine, PG01037, L-741626, L-741742, CGP55845, AM251, and QX-314 were obtained from Tocris Bioscience. JHU37160 was from Hello Bio. Cocaine was from the NIDA Drug Supply Program. All the other chemicals were from Thermo Fisher Scientific or Sigma-Aldrich. Pharmacological inhibition of kinases was done using previously validated inhibitors (*34*, *132*–*135*).

### Quantification and statistical analysis

All data are presented as means ± SEM. Sample size, statistical tests used, and results for all analyses are reported in table S1.

Electrophysiological statistical analyses were performed using GraphPad Prism (v8.0), using parametric *t* tests when applicable, nonparametric Mann-Whitney tests, Wilcoxon matched-pairs signed-rank tests, and Kruskal-Wallis analysis of variance (ANOVA), as appropriate. To compare distributions, a Kolmogorov-Smirnov test was used.

For behavioral studies (Fig. 7 and fig. S5), two-tailed unpaired *t* tests were used to compare two groups, and one-way ANOVA or mixed-effects models with multiple-comparisons corrections were applied when three or more groups were analyzed. Learning trajectories for the RR and SAA tasks were assessed using mixed-effects models in GraphPad Prism (v11.0), with genotype or virus type as a between-subject factor and training day as a within-subject factor. Planned pairwise comparisons for each training day were performed using multiple comparisons test, based on the a priori hypothesis that genotype- or virus-related differences in performance would emerge across later training stages. Adjusted *P* values are reported throughout, with α = 0.05 as the threshold for statistical significance.

Sex was incorporated as a biological variable in the statistical analyses using mixed-effects models or two-way ANOVA for the SAA, OF, and RR tests, as appropriate (fig. S5). Data were analyzed using two- or three-factor mixed-effects models in GraphPad Prism (v11.0) or R (v4.5.2), with training day as a repeated measure and sex (two levels) and virus or phenotype (two to three levels) as between-subject factors. Subject was included as a random intercept. Type III sums of squares were used to evaluate main effects and interactions. For fig. S5N, SAA data were analyzed using linear mixed-effects models fitted by restricted maximum likelihood in R (v4.5.2) using the packages lme4 and lmerTest.

Statistical significance was defined as *P < 0.05, **P < 0.01, and ***P < 0.001. The number of animals (*N*) and the number of cells (*n*) are specified in the figure legends.
