# Supplementary material for: Dopamine D2 receptors bypass canonical signaling to directly tune NMDA receptor function and aversive learning
Source: Sci Adv. 2026 Jul 23;12(30):eaee6579. doi: 10.1126/sciadv.aee6579 (PMC13394467; doi:10.1126/sciadv.aee6579)
Supplement: Supplementary file 1 — Figs. S1 to S5 Table S1 [file sciadv.aee6579_sm.pdf]

Science Advances
AAAS

Supplementary Materials for

Dopamine D2 receptors bypass canonical signaling to directly tune NMDA receptor function and aversive learning

Sheng Gong *et al.*

Corresponding author: Jonathan A. Javitch, jonathan.javitch@nyspi.columbia.edu;
Christopher P. Ford, christopher.ford@cuanschutz.edu

Sci. Adv. **12**, eaee6579 (2026)
DOI: 10.1126/sciadv.aee6579

This PDF file includes:

Figs. S1 to S5
Table S1

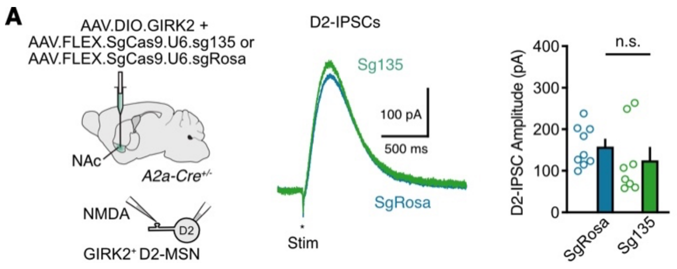

Supplemental Figure 1 (Associated with Figure 2):

(A) Injection of AAV9.DIO.GIRK2 as well as AAV.FLEX.SgCas9.U6.sg135 or AAV.FLEX.SgCas9.U6.sgRosa in A2A-Cre mice. Representative traces and quantification of electrically evoked D2-IPSC. sgRosa: n/N = 9/4. sg135: n/N = 8/4.

Summary data are mean ± SEM. ns = $p > 0.05$. Extended statistical data and tests are provided in Table S1.

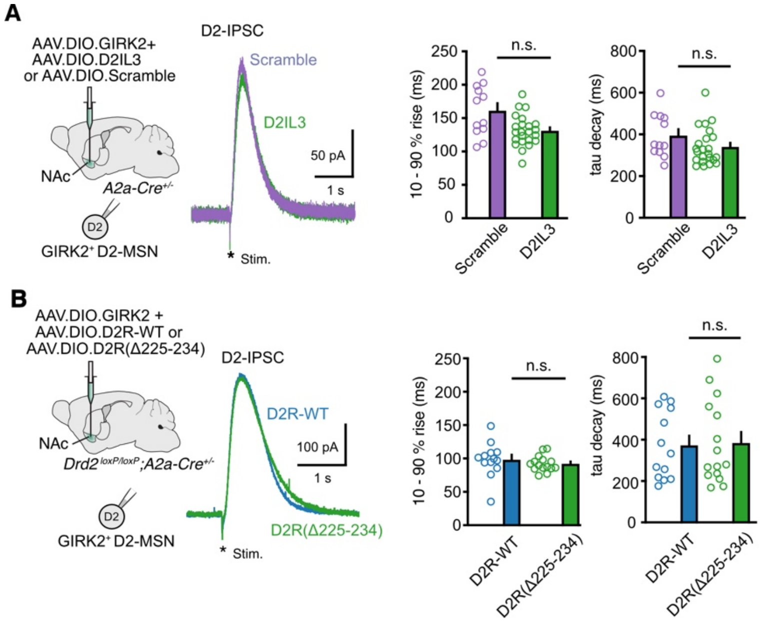

Supplemental Figure 2 (associated with Figure 3)

(A) Injection of AAV.DIO.GIRK2 as well as AAV.DIO.D2IL3 or AAV.DIO.scramble in A2A-Cre mice and recordings of evoked D2-IPSCs from D2-MSNs expressing D2-IL3(n/N = 23/7) or scrambled (n/N = 12/4) sequence illustrating a lack of effect of D2R IL3 on D2-IPSC kinetics.

(B) Injection of AAV.DIO.GIRK2 as well as AAV.DIO.D2R-WT or AAV.DIO.D2R(Δ225-234) in D2R cKO (Drd2$^{loxp/loxp}$; A2a-Cre $^{+/-}$) mice and evoked D2-IPSCs from D2-MSNs expressing D2R-WT(n/N =13/4) or D2R(Δ225-234) (n/N = 15/4) illustrating a lack of effect of D2R IL3 on D2-IPSC kinetics.

Summary data are mean ± SEM. ns = p > 0.05, * = p < 0.05, ** = p < 0.01. Extended statistical data and tests are provided in Table S1.

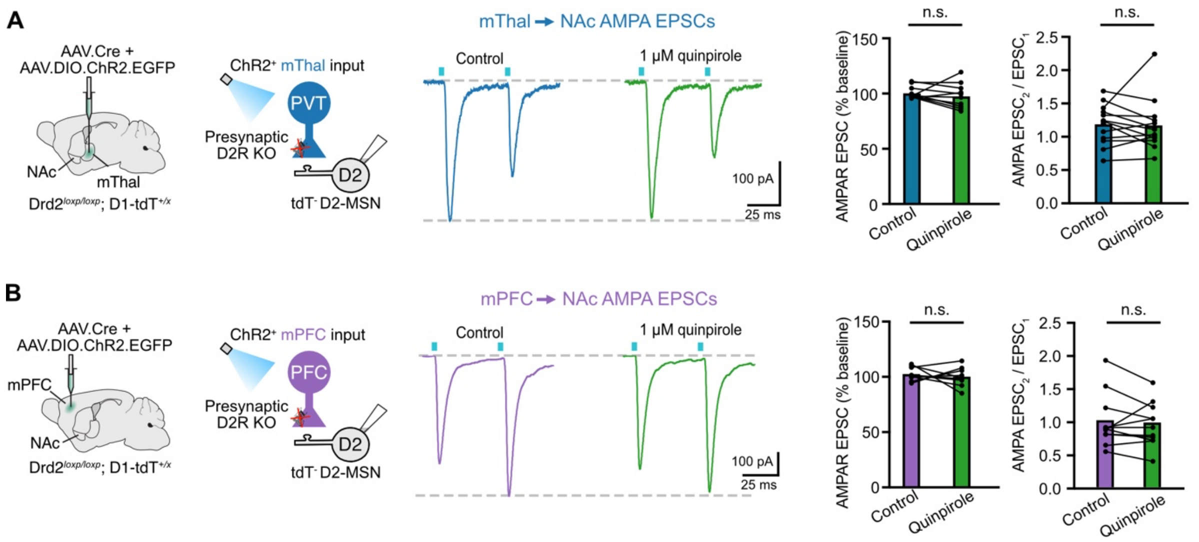

Supplemental Figure 3 (associated with Figure 5)

(A) Midline thalamus (PVT) injection of AAV.Cre and AAV.DIO.ChR2.EGFP in Drd2$^{loxp/loxp}$; D1-tdT $^{+/-}$ mice and evoked AMPA EPSCs showing a lack of effect of quinpirole (1 µM) on EPSC amplitude or paired pulse ratios.

(B) PFC (mPFC) injection of AAV.Cre and AAV.DIO.ChR2.EGFP in Drd2$^{loxp/loxp}$; D1-tdT $^{+/-}$ mice and evoked AMPA EPSCs showing a lack of effect of quinpirole (1 µM) on EPSC amplitude or paired pulse ratios.

Summary data are mean ± SEM. ns = p > 0.05. Extended statistical data and tests are provided in Table S1.

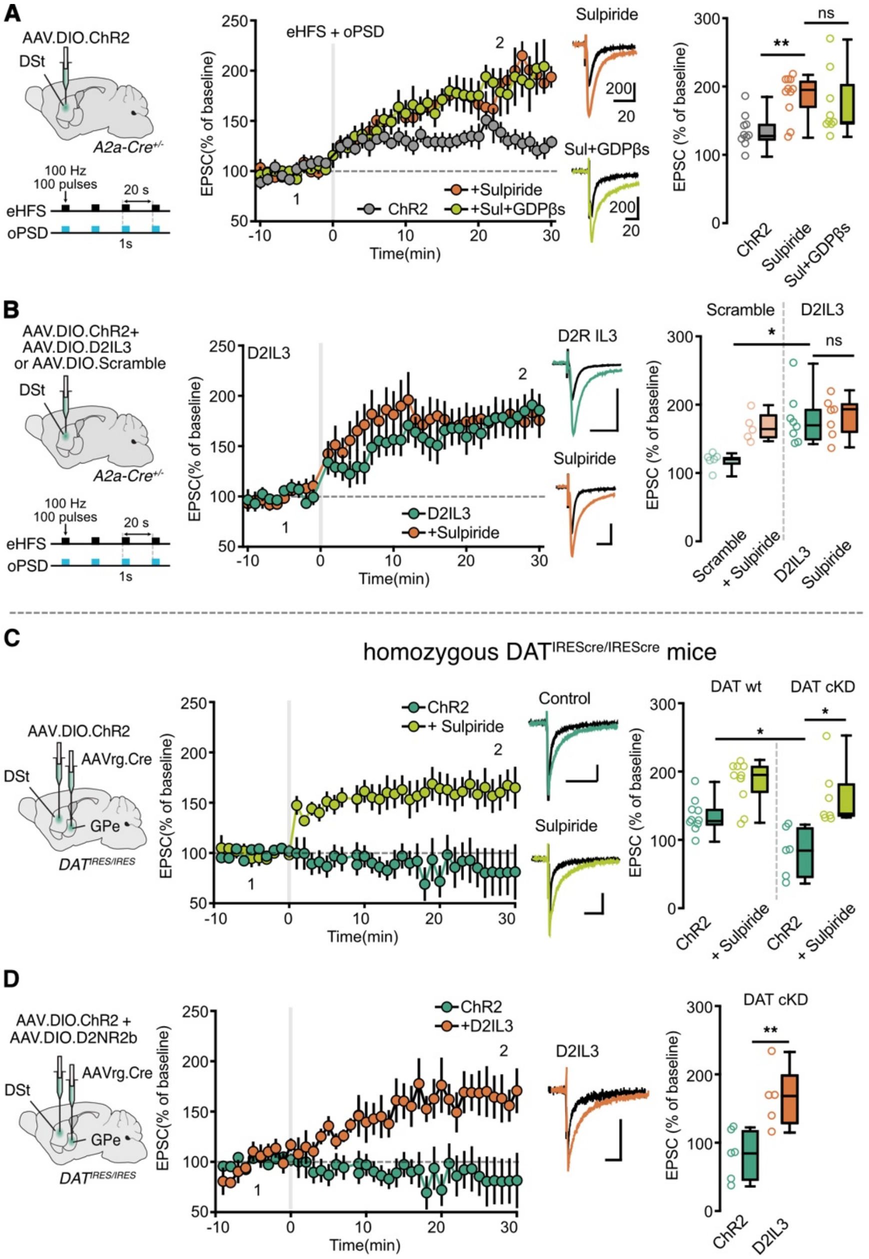

Supplemental Figure 4 (associated with Figure 6)

(A) Left: EPSC (% of baseline) before and after the eHFS + oPSD in the presence of the D2R antagonist sulpiride with (n/N = 9/3) or without (n/N = 11/9) the GDPβs based internal. Middle right: Representative traces with before and after the eHFS+ oPSD protocol. Right: quantification of the EPSC (% of baseline).

(B) Left: Schematic of the injection of AAV.DIO.D2RIL3 or AAV.DIO.scramble with AAV. DIO.ChR2 into the striatum of A2A-Cre mice. Middle: EPSC (% of baseline) before and after the eHFS + oPSD comparing the effect of sulpiride in D2R IL3 or scramble control expressing D2-MSNs. Middle right: Representative traces with before and after the eHFS+ oPSD protocol. Right: quantification of the EPSC (% of baseline). Scramble

control: n/N = 6/5. Scramble sulpiride: n/N = 6/6. D2IL3: n/N = 8/5. D2IL3+sulpiride: n/N = 7/4.

(C) Left: Schematic of the injection of AAVrg.hSyn.Cre into the GPe and AAV.FLEX.ChR2 into the DStr of $DAT^{wt/wt}$ or $DAT^{IREScre/IREScre}$ mice. Middle left: EPSC (% of baseline) before and after the HFS paired with the oPSD protocol with or without sulpiride. Middle right: representative traces of evoked EPSCs. Right: quantification of the EPSC (% of baseline). DAT cKD: n/N = 6/5. DAT cKD sulpiride: n/N = 7/4.

(D) Left: Schematic of the injection of AAVrg.hSyn.Cre into the GPe and AAV.FLEX.ChR2 with AAV.DIO.IL3 into the DStr of $DAT^{IREScre/IREScre}$ mice. Middle left: EPSC (% of baseline) before and after the HFS paired with the oPSD protocol. Middle right: representative traces of evoked EPSCs. Right: quantification of the EPSC (% of baseline). D2IL3: n/N = 5/2.

Summary data are mean ± SEM. ns = $p > 0.05$, * = $p < 0.05$, ** = $p < 0.01$. Extended statistical data and tests are provided in Table S1.

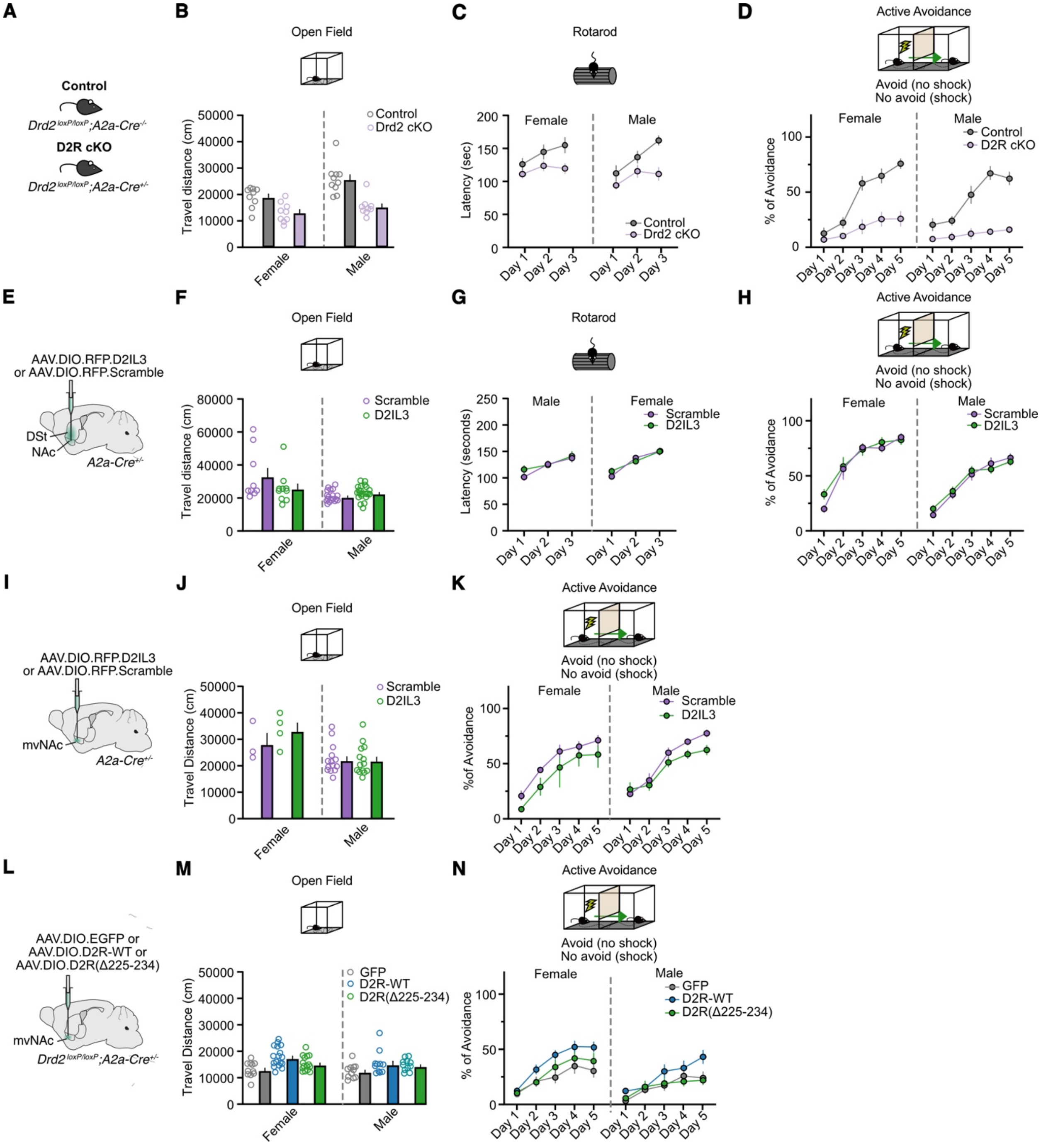

Supplemental Figure 5 (associated with Figure 7). Sex-stratified analyses associated with Figure 7.

Across experiments, we did not detect significant sex × genotype (or sex × viral manipulation) interactions in the shuttle avoidance task or other key behavioral measures, indicating that the effects of disrupting the D2R–GluN2B interaction were not sex-dependent. One exception was the open field test in the broad striatal injection experiment (panel F), in which a significant sex × AAV interaction was detected; however, as no main effect of virus was observed in this experiment, this interaction did

not reflect a sex-dependent effect of the manipulation on locomotion. Data from male and female mice were therefore pooled for the primary analyses in Figure 7.

(A) Control and D2R cKO mice used for behavioral analysis in B-D.

(B) Open field test of control and D2R cKO mice. Control: N=19 (8 females, 11 males); D2R cKO: N=18 (9 females, 9 males). Significant main effects of sex and genotype were observed, but no sex × genotype interaction.

(C) Accelerating rotarod test (3 days) of control and D2R cKO mice. Control: N=19 (8 females, 11 males); D2R cKO: N=18 (9 females, 9 males). A significant effect of time and genotype was observed, with no main effect of sex and no sex-related interactions.

(D) Active avoidance learning (5 days) of control and D2R cKO mice. Control: N=22 (7 females, 15 males); D2R cKO: N=21 (12 females, 9 males). Significant main effects of sex and time, and a sex × time interaction were observed. Genotype significantly affected learning across days with no sex × genotype interaction (E) A2A-Cre mice expressing D2-IL3 minigene or scramble broadly in NAc and DSt (used for behavioral analysis in F-H).

(F) Open field test of mice expressing D2-IL3 minigene or scramble broadly in NAc and DSt. Scramble: N=27 (9 females, 18 males); D2-IL3: N=32 (10 females, 22 males). A significant main effect of sex and sex × AAV interaction was detected, though no main effect of virus was observed.

(G) Accelerating rotarod test (3 days) of mice expressing D2-IL3 minigene or scramble broadly in NAc and DSt. Scramble: N=26 (9 females, 17 males); D2-IL3: N=32 (10 females, 22 males). No effects of sex, virus, or significant interactions were detected.

(H) Active avoidance learning (5 days) of mice expressing D2-IL3 minigene or scramble broadly in NAc and DSt. Scramble: N=26 (9 females, 17 males); D2-IL3: N=32 (10 females, 22 males). A main effect of time and sex was present but no sex × virus interaction was detected.

(I) A2A-Cre mice expressing D2-IL3 minigene or scramble selectively in mvNAc (used for behavioral analysis in J-K).

(J) Open field test of mice expressing D2-IL3 minigene or scramble in mvNAc. Scramble: N=17 (3 females, 14 males); D2-IL3: N=18 (4 females, 14 males). A main effect of sex was present, but no sex × virus interaction was detected.

(K) Active avoidance learning (5 days) of mice expressing D2-IL3 minigene or scramble in mvNAc. Scramble: N=17 (3 females, 14 males); D2-IL3: N=18 (4 females, 14 males). A main effect of time but no sex × virus interaction was detected.

(L) D2R cKO mice expressing GFP, D2R-WT or D2R(Δ225-234) selectively in mvNAc (used for behavioral analysis in M-N).

(M) Open field test of D2R cKO mice expressing GFP, D2R-WT or D2R(Δ225-234) in mvNAc. GFP: N=22 (11 females, 11 males); D2R-WT: N=28 (16 females, 12 males); D2R(Δ225-234): N=26 (15 females, 11 males). A significant main effect of virus was detected with no sex × virus interaction.

(N) Active avoidance learning (5 days) of D2R cKO mice expressing GFP, D2R-WT or D2R(Δ225-234) in mvNAc. GFP: N=23 (12 females, 11 males); D2R-WT: N=29 (17 females, 12 males); D2R(Δ225-234): N=27 (16 females, 11 males). Significant effects of time, sex, and virus were detected, with significant time × sex and time × virus interactions, but no sex × virus interaction, indicating comparable rescue effects across sexes.

Summary data are mean ± SEM. Two-way ANOVA was used for OF. For RR and SAA, a three-factor mixed-effects model was used with time as a repeated measure and sex and virus/genotype as between-subject factors. No post-hoc comparisons were performed. Extended statistical data and tests are provided in Table S1.

Table S1. Extended Statistical Data

Figure	Pannel	Sample Size	Mean ± SEM	p-value/statistical results	Statistical test	Post-hoc test	Post-hoc comparisons between treatment groups	Post-hoc comparisons within treatment groups
**Table S1 Main figures**								
1	A	n = 14, N = 5	control: 370.4 ± 52.3 pA<br>AP5: 42.5 ± 3.5 pA	p = 0.0001	Wilcoxin matched-pairs signed rank test			
	B	n = 7, N = 2	control: 577.4 ± 59.7 pA<br>sgGrin1: 56.5 ± 9.5 pA	p = 0.0001	Mann–Whitney U test			
	D	Dopamine: n = 10, N = 5<br>Quinpirole: n = 7, N = 4<br>SKF38393: n = 6, N = 3	Dopamine: 31.5 ± 1.6%<br>Quinpirole: 29.1 ± 2.9%<br>SKF38393: 4.17 ± 1.3%	H = 10.77<br>p = 0.0016	Kruskal-Wallis ANOVA	Dunn–Šídák multiple test	Adjusted p values:<br>SKF vs. DA: p = 0.0013<br>SKF vs. quin: p = 0.0074	
	E	Ifenprodil: n = 8, N = 4<br>PEAQX: n = 7, N = 4	Ifenprodil: 51 ± 4.4%<br>PEAQX: 36.1 ± 4.0%	p = 0.0319; U = 9.5	Mann–Whitney U test (two-tailed exact)			
	F	Ifenprodil + DA: n = 6, N = 4<br>PEAQX + DA: n = 7, N = 4	Ifenprodil + DA: 5.1 ± 1.2%<br>PEAQX + DA: 29.3 ± 1.6%	H = 13.62<br>p = 0.0011	Kruskal-Wallis ANOVA	Dunn–Šídák multiple test	Adjusted p values:<br>DA vs. Ifenrprodil + DA: p = 0.0005<br>DA vs. PEAQX + DA: p = 0.7261	
	G	DSt: n = 14, N =5<br>NAc: n = 17, N = 5	DSt: 10.2 ± 0.7<br>NAc: 11.6 ± 1.2	p = 0.35	Mann–Whitney U test			
	H	n = 3-8, N = 5	best fit values: amplitude: 22.2 pA mean: 164.4ms	R2 = 0.2154	Nonlinear Gaussian least squares fit			
	I	n = 6, N = 3	2.8 ± 4.3 pA	p < 0.0001	Mann–Whitney U test			
	J	n = 7, N = 4	Control: 552.4 ± 59.0 pA<br>ACh: 543.9 ± 56.5 pA	p = 0.9375	Wilcoxon signed-rank test			
	K	n = 10, N = 3	289.7 ± 45.64 pA					
	L	n = 4, N = 2		p = 0.0396	Wilcoxin matched-pairs signed rank test			
	M	n = 6, N = 2		p = 0.1562	Wilcoxin matched-pairs signed rank test			
2	A	n = 5, N = 2		p = 0.0197	Wilcoxin matched-pairs signed rank test			
	B	Control: n = 10, N = 3<br>GDPbs: n = 8, N = 3	Control: 31.50 ± 1.607%<br>GDPBs: 30.2 ± 1.1%	p = 0.3959; U = 30	Mann–Whitney U test (two-tailed exact)			
	D	D2R-WT: n = 13, N = 4<br>D2R-ARB: n = 12, N = 4	D2R-WT: 337.1 ± 43.8 pA<br>D2-ARB: 34.9 ± 6.9 pA	p < 0.0001	Mann–Whitney U test (two-tailed exact)			
	E	D2R-WT: n = 8, N = 4<br>D2R-ARB: n = 8, N = 4	D2R-WT: 513.2 ± 64.8 pA<br>D2-ARB: 51.9 ± 19.8 pA	p < 0.0001	Mann–Whitney U test (two-tailed exact)			
	F	D2R-WT: n = 12, N = 4<br>D2R-ARB: n = 10, N = 4	D2R-WT: 25 ± 3.9%<br>D2-ARB: 27.8 ± 2.8%	p = 0.4	Mann–Whitney U test (two-tailed exact)			
	G	Rp-cAMP: n = 6, N = 5<br>Rp-cAMP + DA: n = 8, N = 5	Rp-cAMP: 8.5 ± 1.7 %<br>Rp-cAMP + DA: 30.6 ± 1.2 %	Rp-cAMP vs Rp-cAMP + DA: p = 0.0007<br>U = 0	Mann–Whitney U test (two-tailed exact)			
		KT5720: n = 6, N = 4<br>KT5720 + DA: n = 7, N = 4	KT5720: 7.7 ± 2.9%<br>KT5720 + DA: 33.9 ± 1.7%	KT5720 vs KT5720 + DA: p = 0.0012<br>U = 0	Mann–Whitney U test (two-tailed exact)			
		PKI + DA: n = 8, N = 3	PKI + DA: 33.6 ± 2.1%	p > 0.05 (compared with DA)	Mann–Whitney U test			
		H7: n = 7, N = 3<br>H7 + DA: n = 8, N = 3	H7: 10.6 ± 2.6%<br>H7 + DA: 30 ± 2.6%	p > 0.05; Mann-Whitney test (compared with DA)<br>H7 vs H7+DA: p = 0.0006; U = 1	Mann–Whitney U test (two-tailed exact)			
	H	TDZD + DA: n = 7, N = 4<br>SB216763 + DA: n = 8, N = 4	TDZD + DA: 26.1 ± 2.0%,<br>SB216763 + DA: 28.1 ± 2.0%	Test statistic (H): 4.750<br>p = 0.0930	Kruskal-Wallis ANOVA	Dunn–Šídák multiple test	Adjusted p values:<br>control vs TDZD: p = 0.0701<br>control vs SB: p = 0.3212	
	I	sgRosa: n = 8, N = 4<br>sg135: n = 7, N = 4	sgRosa: 55.9 ± 2.5%<br>sg135: 32.61 ± 3.0%	p < 0.0001	Mann–Whitney U test			
	J	sgRosa: n = 11, N = 4<br>sg135: n = 8, N = 4	sgRosa: 31.5 ± 3.8%<br>sg135: 23.7 ± 4.0%	p = 0.252	Mann–Whitney U test (two-tailed exact)			
3	A	Scramble: n = 11, N = 4<br>D2IL3: n = 22, N = 4	Scramble amplitude: 206.5 ± 31.3 pA ; D2IL3 amplitude: 161.2 ± 16.7 pA<br>Scramble rise: 161.2 ± 11.0 ms; D2IL3 rise: 131.1 ± 4.8 ms<br>Scramble decay: 394.3 ± 30.0 ms; D2IL3 decay: 340.6.1 ± 18.4 ms	p = 0.1446<br>U = 68	Mann–Whitney U test (two-tailed exact)			
	B	Scramble: n = 7, N = 4<br>D2IL3: n = 6, N = 4	Scramble: 415.6 ± 40.7 pA<br>D2IL3: 450.0 ± 51.2 pA	p = 0.6282<br>U = 17	Mann–Whitney U test (two-tailed exact)			
	C	Scramble: n = 8, N = 3<br>D2IL3: n = 8, N = 3	sgRosa: 31.5 ± 3.8%<br>sg135: 23.7 ± 4.0%	p = 0.252	Mann–Whitney U test (two-tailed exact)			
	D	Scramble: n = 7, N = 5<br>D2IL3: n = 11, N = 5	Scramble: 23.1 ± 5.0%<br>D2IL3: 1.9 ± 2.9%	p = 0.0085; U = 10.5	Mann–Whitney U test (two-tailed exact)			
	F	D2R-WT: n = 13, N = 4<br>D2R(Δ225-234): n = 15, N = 4	D2R-WT: 382.6 ± 51.2 pA<br>D2R(Δ225-234): 317.7 ± 57.0 pA	p = 0.295	Mann–Whitney U test (two-tailed exact)			
	G	D2R-WT: n = 9, N = 4<br>D2R(Δ225-234): n = 9, N = 4	D2R-WT: 545.9 ± 80.4 pA<br>D2R(Δ225-234): 550.6 ± 68.1 pA	p = 0.730	Mann–Whitney U test (two-tailed exact)			
	H	D2R-WT: n = 12, N = 4<br>D2R(Δ225-234): n = 9, N = 4	D2R-WT: 25.8 ± 3.9 pA<br>D2(Δ225-234): 5.0 ± 4.1 pA	p = 0.0033	Mann–Whitney U test (two-tailed exact)			
4	B	Saline: n = 16, N = 4<br>Cocaine: n = 18, N = 5	Saline: 11.8 ± 0.9 uM<br>Cocaine: 10.2 ± 0.4 uM	EC 50 Sal vs Coc: p = 0.122	Mann–Whitney U test (two-tailed exact)			
	D	DAT wt/wt: n = 13, N = 3<br>DAT cre/cre: n = 11, N = 4	D2-IPSC: DAT wt/wt: 396.8 ± 13.4 ms DAT cre/cre: 561 ± 11.8 ms<br>tau decay: DAT wt/wt: 371.5 ± 12.0 ms DAT cre/cre: 572.3 ± 11.3 ms	D2-IPSC: p < 0.0001<br>tau decay: p < 0.0001	Mann–Whitney U test (two-tailed exact)			
	F	DAT wt/wt: n = 8, N = 2<br>DAT cre/cre: n = 8, N = 3	DAT wt/wt: 31.4 ± 1.7%<br>DAT cre/cre: 39.0 ± 2.0%	p = 0.0081; U = 7.5	Mann-Whitney U, Two-tailed, exact			
5	C	mThal: n = 12, N = 2<br>mPFC: n = 9, N = 3	mThal PPR: 1.062 ± 0.08167<br>mPFC PPR: 0.6919 ± 0.08563	p = 0.0043; U = 15	Mann–Whitney U test (two-tailed exact)			
	E	mThal + DA: n = 15, N = 4<br>mPFC + DA: n = 12, N = 5<br>mThal + Quin: n = 8, N = 2<br>mPFC + Quin: n = 11, N = 6	mThal + DA: 25.44 ± 4.370%<br>mPFC + DA: -1.196 ± 2.901%<br>mThal + Quin: 20.68 ± 3.923%<br>mPFC + Quin: 0.8104 ± 2.825%	mThal + DA vs mPFC + DA: p = 0.0001; U = 19<br>mThal + Quin vs mPFC + Quin: p = 0.0005; U = 5	Mann–Whitney U test (two-tailed exact)			
	F	n = 10, N = 4	DA opto: 2.491 ± 3.552%<br>DA ionto: 25.25 ± 4.911%	p = 0.0098	Wilcoxin matched-pairs signed rank test			
	G	mThal + Ifenprodil: n = 12, N = 4<br>mPFC + Ifenprodil: n = 9, N = 5<br>mThal decay: n = 14, N = 4<br>mPFC decay: n = 11, N = 4	mThal % inhibition: 59.19 ± 2.168%<br>mPFC % inhibition: 55.65 ± 3.478%<br>mThal decay: 47.68 ± 4.247 ms<br>mPFC decay: 44.64 ± 4.568 ms	% inhibition p = 0.6511 U = 47<br>tau decay p = 0.6867	Mann-Whitney U, Two-tailed, exact			
	H	n = 11, N = 4	NMDA EPSC BL: 535.7 ± 124.6 pA<br>NMDA EPSC TBOA: 633.6 ± 134.4 pA<br>decay BL: 52.97 ± 4.88 ms<br>decay TBOA: 60.60 ± 5.487 ms	NMDA EPSC p = 0.0098<br>tau decay p = 0.0020	Wilcoxin matched-pairs signed rank test (both graphs)			
	I	mPFC + DA: n = 6, N = 2<br>mPFC + DA in TBOA: n = 13, N = 5	mPFC + DA: 5.605 ± 4.889 %<br>mPFC + DA in TBOA: 25.20 ± 5.047 %	p = 0.0365; U = 15	Mann–Whitney U test (two-tailed exact)			
6	B	ChR2: n = 10, N = 9<br>mCherry: n = 6, N = 3	ChR2: 135.1 ± 7.1 %<br>mCherry: 96.7 ± 5.7 %	p = 0.001	Mann–Whitney U test (two-tailed exact)			
	D	Sulpiride: n = 11, N = 9	Sulpiride: 178.3 ± 13.3%	p = 0.0011	Mann–Whitney U test (two-tailed exact)			
	E	D2IL3: n = 8, N = 5<br>D2IL3 + Sulpiride: n = 7, N = 4	D2IL3: 178.3 ± 13.3%<br>D2IL3 + Sulpiride: 180.1 ± 10.7%	H = 11.16, p = 0.0038	Kruskal-Wallis ANOVA	Dunn–Šídák multiple test	Adjusted p values:<br>ChR2 vs D2IL3: p = 0.0195<br>ChR2 vs D2IL3 + sulpiride: p = 0.0107	
7	B	Control: N = 19; D2R cKO: N = 18	Control: 22630 ± 1365 cm<br>cKO: 14326 ± 872.4 cm	t(30.34) = 5.126, p < 0.0001	Unpaired t test with Welch's correction (two-tailed)			
	C	Control: N = 19; D2R cKO: N = 18	Control: Day 1: 118.8; Day 2: 140.5; Day 3: 158.7 (s)<br>cKO: Day 1: 102.6; Day 2: 119.5; Day 3: 115.4 (s)	Time: F(1.635,57.23) = 21.32, p < 0.0001;<br>Genotype: F(1,35) = 10.94, p = 0.0022;<br>Interaction: F(1.635,57.23) = 5.954, p = 0.0073	Mixed-effects model (REML), repeated measures (Time × Genotype), Geisser-Greenhouse correction	Tukey's multiple comparisons test	Ctrl vs CKO, Day 1 p>0.05; Day 2 p = 0.0330; Day3, p < 0.0001	Ctrl Day1 vs Day2, p = 0.0004; Ctrl Day1 vs Day3, p < 0.0001; Ctrl Day2 vs Day3, p = 0.0285; CKO Day1 vs Day2, p = 0.0161
	D	Control: N = 22; D2R cKO: N = 21	Control: Day 1: 17.26; Day 2: 23.31; Day 3: 51.92; Day 4: 66.28; Day 5: 67.96 (%)<br>cKO: Day 1: 7.136; Day 2: 9.831; Day 3: 15.86; Day 4: 20.61; Day 5: 21.57 (%)	Time: F(3.125,127.3) = 55.63, p < 0.0001;<br>Genotype: F(1,41) = 47.29, p < 0.0001;<br>Interaction: F(3.125,127.3) = 18.38, p < 0.0001	Mixed-effects model (REML), repeated measures (Time × Genotype), Geisser-Greenhouse correction	Tukey's multiple comparisons test	Between groups: Day 1, p = 0.0267; Day 2, p = 0.0015; Day 3, p < 0.0001; Day 4, p < 0.0001; 5, p < 0.0001.	Control (within): 1 vs 3, p < 0.0001; 1 vs 4, p < 0.0001; 1 vs 5, p < 0.0001; 2 vs 3, p < 0.0001; 2 vs 4, p < 0.0001; 2 vs 5, p < 0.0001; 3 vs 4, p = 0.0318; 3 vs 5, p = 0.0065. CKO (within): 1 vs 4, p = 0.0452; 1 vs 5, p = 0.0382; 2 vs 5, p = 0.0257.
	F	Scramble: N = 27; D2 IL3: N = 32	Scramble: 25306 ± 2064 cm<br>D2 IL3: 24126 ± 1137 cm	t(41.00) = 0.5008, p = 0.6192	Unpaired t test with Welch's correction (two-tailed)	Welch's t-test	test: t(41.00) = 0.5008, p = 0.6192 (ns); F test for variance: F(26,31) = 2.783, p = 0.0071	
	G	Scramble: N = 26; D2 IL3: N = 32	Scramble: Day 1: 102.6; Day 2: 133.8; Day 3: 146.1 (s)<br>D2 IL3: Day 1: 113.9; Day 2: 129.3; Day 3: 147.2 (s)	Time: F(1.587,88.89) = 44.64, p < 0.0001<br>Group: F(1,56) = 0.2612, p = 0.6113<br>Interaction: F(1.587,88.89) = 1.898, p = 0.1639	Mixed-effects model (REML), repeated measures (Time × Group), Geisser-Greenhouse correction	Tukey's multiple comparisons test	no significant pairwise comparisons	
	H	Scramble: N = 26; D2 IL3: N = 32	Scramble: Day 1: 5.077; Day 2: 12.62; Day 3: 18.31; Day 4: 20.42; Day 5: 22.08 (%)<br>D2 IL3: Day 1: 7.531; Day 2: 13.25; Day 3: 18.25; Day 4: 19.59; Day 5: 20.97 (%)	Time: F(2.896,162.2) = 147.8, p < 0.0001<br>Virus: F(1,56) = 0.0288, p = 0.8659<br>Interaction: F(2.896,162.2) = 1.933, p = 0.1285	Mixed-effects model (REML), repeated measures (Time × Virus), Geisser-Greenhouse correction	Tukey's multiple comparisons test	Between groups: Day1, p = 0.0134. Day 2-5, p>0.05.	A2ACre+Scramble (within): 1 vs 2, p < 0.0001; 1 vs 3, p < 0.0001; 1 vs 4, p < 0.0001; 1 vs 5, p < 0.0001; 2 vs 3, p = 0.0027; 2 vs 4, p = 0.0003; 2 vs 5, p < 0.0001; 3 vs 5, p < 0.0001. A1Acre+Minigene (within): 1 vs 2, p = 0.0003; 1 vs 3, p < 0.0001; 1 vs 4, p < 0.0001; 1 vs 5, p < 0.0001; 2 vs 3, p < 0.0001; 2 vs 4, p < 0.0001; 2 vs 5, p < 0.0001; 3 vs 5, p = 0.0106.

	J	Scramble: N = 17; D2 IL3: N = 18	Scramble: 23473 ± 1477 cm<br>D2 IL3: 24709 ± 1738 cm	t(32.44) = 0.5418, p = 0.5916	Unpaired t test with Welch's correction (two-tailed)	Welch's t-test	test: t(32.44) = 0.5418, p = 0.5916 (ns); F test for variance: F(17,16) = 1.466, p = 0.4493	
	K	Scramble: N = 17; D2 IL3: N = 18	Scramble: Day 1: 6.706; Day 2: 11.00; Day 3: 18.06; Day 4: 20.76; Day 5: 22.94 (%)<br>D2 IL3: Day 1: 6.833; Day 2: 9.056; Day 3: 15.06; Day 4: 17.50; Day 5: 18.44 (%)	Time: F(2.752,90.82) = 77.99, p < 0.0001<br>Virus: F(1,33) = 3.932, p = 0.0558<br>Interaction: F(2.752,90.82) = 1.633, p = 0.1909	Mixed-effects model (REML), repeated measures (Time × Virus), Geisser-Greenhouse correction	Tukey's multiple comparisons test	Between groups: Day 1-3, p>0.05; Day4, p = 0.0256; Day5, p = 0.0082.	Scramble (within): 1 vs 3, p < 0.0001; 1 vs 4, p < 0.0001; 1 vs 5, p < 0.0001; 2 vs 3, p < 0.0001; 2 vs 4, p < 0.0001; 2 vs 5, p < 0.0001; 3 vs 5, p = 0.0004. Minigene (within): 1 vs 3, p = 0.0133; 1 vs 4, p = 0.0003; 1 vs 5, p = 0.0002; 2 vs 3, p = 0.0090; 2 vs 4, p < 0.0001; 2 vs 5, p < 0.0001; 3 vs 5, p = 0.0204.
	M	GFP: N = 22<br>D2R-WT: N = 28<br>D2R(Δ225-234): N = 26	GFP: 12850 ± 584.3 cm<br>D2R-WT: 16804 ± 781.4 cm<br>D2RΔ: 15020 ± 494.2 cm	F(2,73) = 9.038, p = 0.0003	One-way ANOVA (3 groups)	Tukey's multiple comparisons test	GFP+ vs WT+, p = 0.0002	
	N	GFP: N = 23<br>D2R-WT: N = 29<br>D2R(Δ225-234): N = 27	GFP: Day 1: 7.101; Day 2: 17.54; Day 3: 21.59; Day 4: 31.01; Day 5: 27.83 (%)<br>D2R-WT: Day 1: 12.87; Day 2: 25.17; Day 3: 39.08; Day 4: 44.71; Day 5: 48.62 (%)<br>D2RΔ: Day 1: 9.012; Day 2: 19.01; Day 3: 28.40; Day 4: 33.83; Day 5: 32.72 (%)	Time: F(2.925,222.3) = 70.95, p < 0.0001<br>AAV: F(2,76) = 5.417, p = 0.0063<br>Interaction: F(8,304) = 2.058, p = 0.0397	Mixed-effects model (REML), repeated measures (Time × AAV), Geisser-Greenhouse correction	Tukey's multiple comparisons test	Between groups: Day1 GFP vs WT, p = 0.0422; Day3 GFP vs WT, p = 0.0039; Day5 GFP vs WT, p = 0.0016; Day5 WT vs dD2-NR2b, p = 0.0293.	CKO+GFP (within): 1 vs 2, p = 0.0003; 1 vs 3, p = 0.0007; 1 vs 4, p < 0.0001; 1 vs 5, p = 0.0001; 2 vs 4, p = 0.0046; 2 vs 5, p = 0.0246; 3 vs 4, p = 0.0020. CKO+WT D2 (within): 1 vs 2, p = 0.0162; 1 vs 3, p < 0.0001; 1 vs 4, p < 0.0001; 1 vs 5, p < 0.0001; 2 vs 3, p < 0.0001; 2 vs 4, p = 0.0001; 2 vs 5, p < 0.0001; 3 vs 5, p = 0.0272. CKO+dD2-NR2b (within): 1 vs 2, p = 0.0218; 1 vs 3, p = 0.0004; 1 vs 4, p < 0.0001; 1 vs 5, p < 0.0001; 2 vs 3, p = 0.0460; 2 vs 4, p = 0.0048; 2 vs 5, p = 0.0039.
**Table S1 Supplemental figures**								
**S1**	A	sgRosa: n = 9, N = 4<br>sg135: n = 8, N = 4	sgRosa: 158.4 ± 16.2 pA<br>sg135: 125.1 ± 29.6 pA	p = 0.267	Mann–Whitney U test (two-tailed exact)			
**S2**	A	D2-IL3: n = 23, N = 7<br>Scramble: n = 12, N = 4	Scramble rise time: 64.0 ± 2.1 pA; D2IL3 rise time : 61.1 ± 3.9 pA<br>Scramble decay: 340.6 ± 18.4 pA; D2IL3 rise time: 394.3 ± 29.9 pA	p = 0.4<br>p = 0.09	Mann–Whitney U test (two-tailed exact) for both			
	B	D2R-WT: n = 13, N = 4<br>D2R(Δ225-234): n = 15, N = 4	D2-WT rise time: 98.3 ± 7.2 pA; D2(Δ225-234) rise time: 92.2 ± 3.0 pA<br>D2-WT decay: 373.3 ± 45.9 pA; D2(Δ225-234) decay: 384.6 ± 52.2 pA	p = 0.185<br>p= 0.893	Mann–Whitney U test (two-tailed exact) for both			
**S3**	A	n = 16; N = 2	AMPAR EPSC BL: 100.9 ± 1.797 pA<br>AMPAR EPSC Quin: 98.06 ± 3.556 pA<br>AMPAR EPSC2/EPSC1 BL: 1.191 ± 0.08249 pA<br>AMPAR EPSC2/EPSC1 Quin: 1.174 ± 0.1073 pA	AMPAR EPSC: p = 0.1934<br>AMPAR EPSC2/EPSC1: p = 0.4549	Wilcoxin matched-pairs signed rank test			
	B	n = 11; N = 4	AMPAR EPSC BL: 102.2 ± 1.217 pA<br>AMPAR EPSC Quin: 100.6 ± 3.506 pA<br>AMPAR EPSC2/EPSC1 BL: 1.033 ± 0.1423 pA<br>AMPAR EPSC2/EPSC1 Quin: 0.9804 ± 0.1192 pA	AMPAR EPSC: p = 0.7002<br>AMPAR EPSC2/EPSC1: p = 0.3008	Wilcoxin matched-pairs signed rank test			
**S4**	A	Sulpiride + GDPbS: n = 9, N = 3<br>Sulpiride: n = 11, N = 9	Sulpiride + GDPbs: 166.2 ± 11.4%	H = 11.14, p = 0.0038	Kruskal-Wallis ANOVA test	Dunn's multiple test	Adjusted p values:<br>chR2 vs sulpiride: p = 0.0031<br>sulpiride vs sulpiride +GDPbs: p = 0.8899	
	B	Control: n = 6, N = 5<br>Scramble + Sulpiride: n = 6, N = 6<br>D2IL3: n = 8, N = 5<br>D2IL3 + Sulpiride: n = 7, N = 4	Scramble: 117.3 ± 4.8%<br>Scramble + Sulpiride: 168.7 ± 7.6%	Scramble vs D2IL3: H = 13.9, p = 0.003	Kruskal-Wallis ANOVA test	Dunn's multiple test	Adjusted p values:<br>scramble vs D2IL3: p = 0.012<br>D2IL3 vs D2IL3+sulpiride: p >0.9999	
	C	DAT cKD: n = 6, N = 5<br>DAT cKD Sulpiride: n = 7, N = 4	DAT cKD: ChR2: 81.9 ± 14.7%<br>ChR2+ Sulpiride: 162.8 ± 16.6%	H = 20.27 p = 0.0001	Kruskal-Wallis ANOVA test	Dunn's multiple test	Adjusted p values:<br>DAT cKD ChR2 vs +sulpiride: p = 0.017<br>DAT wt ChR2 vs DAT cKD ChR2: p = 0.03	
	D	D2IL3: n = 5, N = 2	164.8 ± 19.8%	p =0.0047	Mann–Whitney U test (two-tailed exact)			
**S5**	B	Control: N = 19 (8 females, 11 males)<br>D2R cKO: N = 18 (9 females, 9 males)	Male Control: 25838 cm<br>Male cKO: 15432 cm<br>Female Control: 19067 cm<br>Female cKO: 13219 cm	Sex: F(1,33)=9.739, p=0.0037<br>Genotype: F(1,33)=31.87, p<0.0001<br>Interaction: F(1,33)=2.506, p=0.1230	Two-way ANOVA (ordinary)			
	C	Control: N = 19 (8 females, 11 males)<br>D2R cKO: N = 18 (9 females, 9 males)	Male Control: Day 1: 112.2; Day 2: 136.8; Day 3: 162.0 (s)<br>Male cKO: Day 1: 94.15; Day 2: 115.5; Day 3: 111.3 (s)<br>Female Control: Day 1: 126.1; Day 2: 144.7; Day 3: 154.9 (s)<br>Female cKO: 111.0; Day 2: 123.6; Day 3: 119.5 (s)	Time: F(2,66) = 20.96, p < 0.0001; Sex, F(1,33) = 0.9385, p = 0.3397; Genotype, F(1,33) = 10.74, p = 0.0025; Time × Sex interaction, F(2,66) = 1.584, p = 0.2128; Time × Genotype interaction, F(2,66) = 5.729, p = 0.0051; Sex × Genotype interaction, F(1,33) = 0.1400, p = 0.7106; Time × Sex × Genotype), F(2,66) = 0.4629, p = 0.6315	Mixed-effects model (REML), repeated measures (GG correction)			
	D	Control: N = 22 (7 females, 15 males)<br>D2R cKO: N = 21 (12 females, 9 males)	Male Control: Day 1: 20.49; Day 2: 24.08; Day 3: 47.64; Day 4: 67.43; Day 5: 62.50 (%)<br>Male cKO: Day 1: 7.400; Day 2: 9.250; Day 3: 12.21; Day 4: 14.06; Day 5: 15.91 (%)<br>Female Control: Day 1: 12.58; Day 2: 22.20; Day 3: 58.09; Day 4: 64.75; Day 5: 75.85 (%)<br>Female cKO: Day 1: 6.938; Day 2: 10.27; Day 3: 18.59; Day 4: 25.53; Day 5: 25.81 (%)	Time: F(4,155)=57.00, p<0.0001; Sex: F(1,39)=0.8616, p=0.3590; Genotype: F(1,39)=46.94, p<0.0001; Time × Sex: F(4,155)=2.609, p=0.0377; Time × Genotype: F(4,155)=20.73, p<0.0001; Genotype × Sex: F(1,39)=0.1107, p=0.7412; Time × Genotype×Sex: F(4,155)=0.7562, p=0.5553.	Mixed-effects model (REML), matching by row factor			
	F	Scramble: N = 27 (9 females, 18 males)<br>D2 IL3: N = 32 (10 females, 22 males)	Male Scramble: 21170 cm<br>Male D2 IL3: 23211 cm<br>Female Scramble: 33578 cm<br>Female D2 IL3: 26141 cm	Interaction: F(1,55)=4.812, p=0.0325<br>Sex: F(1,55)=12.60, p=0.0008<br>AAV: F(1,55)=1.561, p=0.2169	Two-way ANOVA (ordinary)			
	G	Scramble: N = 26 (9 females, 17 males)<br>D2 IL3: N = 32 (10 females, 22 males)	Male Scramble: Day 1: 103.1; Day 2: 138.0; Day 3: 150.7 (s)<br>Male D2 IL3: Day 1: 113.0; Day 2: 131.5; Day 3: 150.0 (s)<br>Female Scramble: Day 1: 101.6; Day 2: 125.7; Day 3: 137.3 (s)<br>Female D2 IL3: Day 1: 115.9; Day 2: 124.5; Day 3: 141.2 (s)	Time: F(2,108)=34.74, p<0.0001; Sex: F(1,54)=1.439, p=0.2356; Virus: F(1,54)=0.3416, p=0.5613; Time × Sex: F(2,108)=1.062, p=0.3495; Time × virus: F(2,108)=1.704, p=0.1869; Sex × virus: F(1,54)=0.1836, p=0.6700; Time × Sex × virus: F(2,108)=0.001269, p=0.9987.	Mixed-effects model (REML), matching by Time			
	H	Scramble: N = 26 (9 females, 17 males)<br>D2 IL3: N = 32 (10 females, 22 males)	Male Scramble: Day 1: 15.07; Day 2: 33.48; Day 3: 51.53; Day 4: 61.34; Day 5: 66.42 (%)<br>Male D2 IL3: Day 1: 20.67; Day 2: 36.49; Day 3: 54.81; Day 4: 56.19; Day 5: 62.99 (%)<br>Female Scramble: Day 1: 19.98; Day 2: 56.24; Day 3: 75.85; Day 4: 75.11; Day 5: 85.10 (%)<br>Female D2 IL3: Day 1: 33.30; Day 2: 58.61; Day 3: 73.93; Day 4: 80.59; Day 5: 82.58 (%)	Time: F(4,232)=141.7, p<0.0001; Sex: F(1,58)=20.51, p<0.0001; virus: F(1,58)=0.2479, p=0.6205; Time × Sex: F(4,232)=2.408, p=0.0502; Time × virus: F(4,232)=1.711, p=0.1482; Sex × virus: F(1,58)=0.1110, p=0.7402; Time × Sex × virus: F(4,232)=0.8201, p=0.5135.	Mixed-effects model (REML), matching by Time			
	J	Scramble: N = 17 (3 females, 14 males)<br>D2 IL3: N = 18 (4 females, 14 males)	Male Scramble: 22401 cm<br>Male D2 IL3: 22216 cm<br>Female Scramble: 28477 cm<br>Female D2 IL3: 33437 cm	Interaction: F(1,31)=1.092, p=0.3040<br>Sex: F(1,31)=12.34, p=0.0014<br>Virus: F(1,31)=0.9404, p=0.3397	Two-way ANOVA (ordinary)			
	K	Scramble: N = 17 (3 females, 14 males)<br>D2 IL3: N = 18 (4 females, 14 males)	Male Scramble: Day 1: 22.60; Day 2: 34.97; Day 3: 59.94; Day 4: 69.93; Day 5: 77.54 (%)<br>Male D2 IL3: Day 1: 6.643; Day 2: 30.45; Day 3: 51.14; Day 4: 58.51; Day 5: 62.32 (%)<br>Female Scramble: Day 1: 21.09; Day 2: 44.40; Day 3: 61.05; Day 4: 65.49; Day 5: 71.04 (%)<br>Female D2 IL3: Day 1: 9.157; Day 2: 29.14; Day 3: 46.62; Day 4: 57.44; Day 5: 58.28 (%)	Time: F(4,124)=48.42, p<0.0001; Sex: F(1,31)=0.3109, p=0.5811; Virus: F(1,31)=3.270, p=0.0803; Time × Sex: F(4,124)=0.7467, p=0.5620; Time × Virus: F(4,124)=0.4163, p=0.7966; Sex × Virus: F(1,31)=0.2378, p=0.6293; Time × Sex×Virus: F(4,124)=0.5264, p=0.7165.	Mixed-effects model (REML), matching by Time			
	M	GFP: N = 22 (11 females, 11 males)<br>D2R-WT: N = 28 (16 females, 12 males)<br>D2R(Δ225-234): N = 26 (15 females, 11 males)	GFP: Female: 13181 cm; Male: 12519 cm<br>D2R-WT: Female: 17745 cm; Male: 15349 cm<br>D2R(Δ225-234): Female: 15261 cm; Male: 14692 cm	Interaction: F(2,70)=0.6486, p=0.5259<br>Sex: F(1,70)=2.561, p=0.1140<br>Virus: F(2,70)=7.859, p=0.0008	Two-way ANOVA (ordinary)			
	N	GFP: N = 23 (12 females, 11 males)<br>D2R-WT: N = 29 (17 females, 12 males)<br>D2R(Δ225-234): N = 27 (16 females, 11 males)	Female GFP: Day 1: 9.990; Day 2: 20.81; Day 3: 24.98; Day 4: 35.24; Day 5: 30.80 (%)<br>Female D2R-WT: Day 1: 12.93; Day 2: 31.73; Day 3: 45.05; Day 4: 52.50; Day 5: 52.10 (%)<br>Female D2RΔ: Day 1: 10.82; Day 2: 20.60; Day 3: 34.34; Day 4: 42.25; Day 5: 39.75 (%)<br>Male GFP:Day 1: 3.935; Day 2: 13.93; Day 3: 17.86; Day 4: 26.34; Day 5: 24.52 (%)<br>Male D2R-WT: Day 1: 12.77; Day 2: 15.82; Day 3: 30.53; Day 4: 33.58; Day 5: 43.57 (%)<br>Male D2RΔ: Day 1: 6.357; Day 2: 16.65; Day 3: 19.68; Day 4: 21.49; Day 5: 22.40 (%)	Time: F(4,292)=67.3708, p<0.0001; Sex: F(1,73)=9.9773, p=0.0023; Virus: F(2,73)=5.3579, p=0.0067; Sex × Virus: F(2,73)=0.2383, p=0.7886; Sex × Time: F(4,292)=2.9237, p=0.0214; Virus × Time: F(8,292)=2.1288, p=0.0332; Sex × Treatment × Day: F(8,292)=1.1680, p=0.3183.	Linear mixed-effects model (REML), Satterthwaite method; Score ~ Sex * Treatment * Day (categorical) + (1|MouseID)			
